# Dual‐Enhanced SERS Satellite Immuno‐Nanocomplex for Multiple PSA‐Mediated PHI Assay Toward Clinical Prostate Cancer Screening

**DOI:** 10.1002/advs.202411747

**Published:** 2024-12-10

**Authors:** Dong Chen, Yilin Ma, Annan Yang, Liping Hu, Hanlin Zhou, Jun Xu, Shanze Chen, Dingmeng Nie, Weifeng Feng, Huaihong Cai, Yanguang Cong, Jiang Pi, Lang Rao, Xueqin Huang, Pinghua Sun, Haibo Zhou

**Affiliations:** ^1^ Institute for Safflower Industry Research Key Laboratory of Xinjiang Phytomedicine Resource and Utilization (Ministry of Education) School of Pharmacy Shihezi University Shihezi 832003 China; ^2^ Department of Urology State Key Laboratory of Oncology in South China Sun Yat‐sen University Cancer Center Guangzhou 510060 China; ^3^ College of Pharmacy The Second Clinical Medical College (Shenzhen People's Hospital), The Fifth Affiliated Hospital Jinan University Guangzhou 510632 China; ^4^ Institute of Chemical Biology Shenzhen Bay Laboratory Shenzhen 518132 China; ^5^ The First Affiliated Hospital of Jinan University Guangzhou 510632 China; ^6^ College of Chemistry and Materials Science Jinan University Guangzhou 510632 China; ^7^ Guangdong Provincial Key Laboratory of Medical Molecular Diagnostics The First Dongguan Affiliated Hospital School of Medical Technology Guangdong Medical University Dongguan 523000 China

**Keywords:** intra‐nanogap particles, PCa screening, prostate health index, prostate specific antigen, surface‐enhanced raman scattering

## Abstract

Prostate specific antigen (PSA) is widely used in liquid biopsy of prostate cancer (PCa) but still faces challenges due to the poor specificity. Herein, this study reports a double‐SERS satellite immunoassay, made of an Au–Ag dealloyed intra‐nanogap nanoflower (Au–Ag DINF) with strong SERS signals and Au magnetic nanoparticles (AuMNPs) with magnetic capture and SERS amplification, for sensing multiple PSA (free PSA (fPSA), complexed PSA (cPSA) and [‐2]proPSA (p2PSA)) toward potential PCa screening. Unlike the previous studies focus on the tPSA and fPSA/tPSA ratio (f/t PSA%), this work introduces a multiple PSA‐mediated Prostate Health Index (PHI) assay with significantly increased the predictive accuracy and specificity of PCa, especially the patients with a tPSA level in the “diagnostic gray zone”. Practical analysis of clinical samples demonstrated the SERS immunoassay is capable of identifying the PCa subjects from healthy candidates (*N* = 13), and monitoring PCa patients before and after surgery (*N* = 5), as well as screening suspicious ones who are in the “gray zone” (*N* = 10). Overall, benefiting from the PSA‐responsive satellite immuno‐nanoassemble strategy, dual‐SERS magnification effect, and multiple PSA‐based PHI assessment, this proposes bioassay held enormous potential for the early diagnosis, screening, monitoring, and prognosis of PCa.

## Graphical abstract

This study reports a double‐SERS satellite nanocomplex immunoassay that made of an Au–Ag dealloyed intra‐nanogap nanoflower (Au–Ag DINF) and Au magnetic nanoparticles (AuMNPs) for multicomponent PSA, and introduces a prostate Health index (PHI) assay for potential PCa screening, especially the patients with a tPSA level in “diagnostic gray zone”.

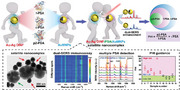

## 1 Introduction

Prostate cancer (PCa) is the most common malignancy affecting male health worldwide.^[^ ^1^ ^]^ Given this, early diagnosis followed by earlier stage PCa treatment is conducive to reducing mortality yet is still challenging due to the lack of available biomarkers for screening. Despite PSA known as tPSA is the widely accepted biomarker for PCa, the overexpression level of which is also associated with other non‐cancerous prostate diseases, such as benign prostatic hyperplasia (BPH) and prostatitis.^[^ ^2^, ^3^ ^]^ Consequently, it is hard for prostatologists to give an accurate diagnosis solely relying on elevated tPSA level, especially in the “diagnostic gray zone” of 4–10 ng mL^−1^.^[^ ^4^ ^]^ Increasing attention has been paid to differentiating the different subtypes of PSA in serum samples to reduce the false positive, from which most of PSA protein binds to serum protease inhibitor α1‐antichymotrypsin called cPSA, while a small portion exists as fPSA in serum. From a clinical point of view, the f/t PSA% improves the specificity of PCa testing to some extent, whereas it still cannot meet the clinical requirements for precise diagnosis, resulting in considerable unnecessary prostate biopsies. Fortunately, the PRO‐PSA Multicentric European Study Group (PROMETHEUS) recently confirmed that p2PSA derivative, namely, PHI exhibited the strongest predictive ability for PCa at initial biopsy in patients whose tPSA level falling in the “diagnostic gray zone”.^[^ ^5^, ^6^ ^]^ In recent years, many methods have been developed for the detection of PSA, such as enzyme linked immunosorbent assay (ELISA), electrochemical immunoassay or chemiluminescence immunoassay,^[^ ^7^, ^8^, ^9^, ^10^ ^]^ while the common issues that are often encountered included low sensitivity, long procedural times, and limited single‐component testing. Accordingly, developing a novel method that allows for sensitive, multi‐component, point‐of‐care and reliable detections toward different isoforms of PSA remains a matter of urgency.

Currently, surface‐enhanced Raman scattering (SERS) has emerged as a promising technique due to its high sensitivity, characteristic fingerprints and multiplexing potential, etc.^[^ ^11^, ^12^, ^13^, ^14^ ^]^ Benefiting from the excitation of local surface plasma resonance (LSPR), noble metallic nanoparticles (NPs) can generate an intensely enhanced local electromagnetic (EM) field (“hot spots”), in which Raman scattering enhancement factors (EF) were magnified up to 1 × 10^8^, even achieving single‐molecule‐level sensitivity.^[^ ^15^, ^16^, ^17^ ^]^ Conventional method usually adsorbed Raman reporter molecules on the surface of SERS probe for SERS enhancement, while these labeling molecules are easily diffused from the SERS substrate and exchanged with other Raman dyes, which is a major barrier for producing reproducible and reliable SERS signals.^[^ ^18^, ^19^ ^]^ Although this problem can be addressed by functionalizing a layer of biomolecules (such as bovine serum, polymers, liposomes, and SiO_2_ nanolayers) as a protective shell on the naked SERS probe surface,^[^ ^20^ ^]^ the SERS measurement would be affected by the photopermeability, thickness and dispersibility of coating, which in turn affect the sensitivity of assay. One fascinating technique is to construct “core‐gap‐shell” SERS enhanced substrates, where Raman molecules are packaged in the nano‐gap that is composed of internal metallic core and external metallic shell, thus producing the intense “hot spots” while avoiding the leakage of Raman molecules. However, how to control the gap morphology and space of SERS substrates for accurately placing Raman molecules within the nanogap remains a challenge. In the previous study, our group has developed Au nano‐bridged nanogaps particles that were constructed with Au core‐Au shell NPs with a uniform intragap (≈2 nm) through thiolated DNA mediation, acquiring highly enhanced and stable SERS signal.^[^ ^21^ ^]^ Unfortunately, some issues that are encountered include complicated synthesis process, low productivity, time and cost, particle stability, and limited reaction conditions (including temperature). In particular, interlayered small molecules (such as thiolated DNA, chiral amino acid, etc.) that occupied contact sites of Raman tags affect the storage and distribution of Raman labels in the gap, which is not conducive to the SERS signals amplification.^[^ ^22^, ^23^ ^]^ Accordingly, new methodologies are needed to construct intragap nanostructures in very high controllability and yield with the desired plasmonic characteristics and functions.

**Scheme 1:**
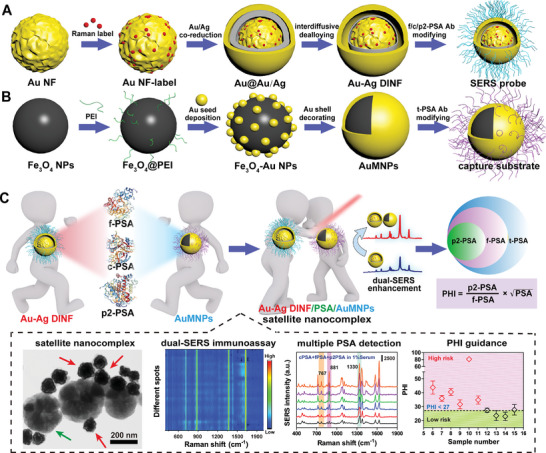
Schematic illustration of the fabricated process for A) Au–Ag DINF (SERS probe) and B) AuMNPs (capture substrate). C) Principle of dual‐enhanced SERS satellite strategy for multicomponent PSA detection toward PHI‐guided PCa diagnosis.

In this work, we report a straightforward, facile selective‐interdiffusive dealloying (SID) chemistry for fabricating Au–Ag DINF without the assistance of interlayer materials. The enhanced EM field generated in the nanometer‐scale interior gap emitted strong, stable and reproducible SERS signals for highly sensitive sensing of PSA. Additionally, AuMNPs with magnetic capture and SERS amplification properties were developed to significantly improve the accuracy and anti‐interference of PSA assay, irrespective of complex interference in the human serum. In the presence of the target PSA, Au–Ag DINF (SERS probe) was assembled with the PSA to form Au–Ag DINF/PSA complex, followed by magnetic capture by AuMNPs (capture substrate) to obtain the magnetic‐plasmonic dual‐SERS satellite nanoassemblies, in which the activated “hot spots” between adjacent Au–Ag DINF and AuMNPs further magnified the SERS enhancement effect for highly quantitative and sensitive PSA detection. Since the multianalyte analysis capability of SERS, multiple PSA simultaneous detection are implemented by encoding three different Raman reports ((4‐nitrothiophenol (4‐NTP), 2,3,5,6‐Tetrafluorobenzenethiol (TFTP), 2‐naphthalenethiol (2‐NT)) corresponding to PSA indicators (fPSA, cPSA, p2PSA) and by establishing a signal‐concentration linear relationship. More importantly, the multiple PSA‐mediated PHI calculation introduced in this study found an increased predictive value when compared with the previous tPSA and f/t PSA%, which is expected to improve the accuracy and specificity of PCa diagnosis. Based on the notable performances, the dual‐SERS based PHI assay can differentiate the PCa subjects whose tPSA level in “diagnostic gray zone”, as well as successfully monitor the PCa patients before and after surgery and assist the f/t PSA% for assessing PCa‐positive/nagative patient. In all patients’ analysis, the PHI demonstrated the best performance (AUC = 0.815) for detection of PCa compared with f/t PSA% (AUC = 0.704), showing the superior diagnostic value especially in the “gray area” of tPSA. Therefore, by incorporating the merits of dual‐enhanced SERS sensing, PSA‐specific satellite strategy, and PHI‐guided analysis, this study provides a novel avenue for early diagnosis, monitoring, and prognosis of PCa, with the aim of reducing unnecessary biopsies (**Scheme** **1**).

## 2 Results and Discussion

### 2.1 Preparation and Characterization of Au–Ag DINF

In this study, Au–Ag DINF with strong, stable, and reproducible SERS signals were synthesized in a high yield (≈95%) by the Au/Ag co‐reduction and selective‐interdiffusive dealloying process, enabling it suitable for highly sensitive PSA‐specific sensing. The Au nanoflower (Au NF) with nanoscale‐tip surfaces was introduced as the core, which was conducive to the formation of “hot spot” (Figure S1, Supporting Information). Further, Au/Ag‐alloyed shell was covered on the surface of Raman label (4‐NTP)‐modified Au NF cores (≈80 nm) by the simultaneous reduction of HAuCl_4_ and AgNO_3_, eventually forming Au@Au/Ag accompanied with the slight red‐shift of the absorption peaks and increase of particles size (Figures S2 and S3, Supporting Information). During the alloy shell formation process, the strong affinity between Ag and the pyrrolidone groups of polyvinylpyrrolidone (PVP) resulted in the faster reduction of Ag precursor as compared to the case of the Au precursor, while Au atoms deposited on the initial Ag layer were further reduced and epitaxial growth. As a result, the space occupied by Ag atoms cannot be filled by Au atoms, leading to an uneven distribution of Au and Ag atoms in the alloy shell.

**Figure 1:**
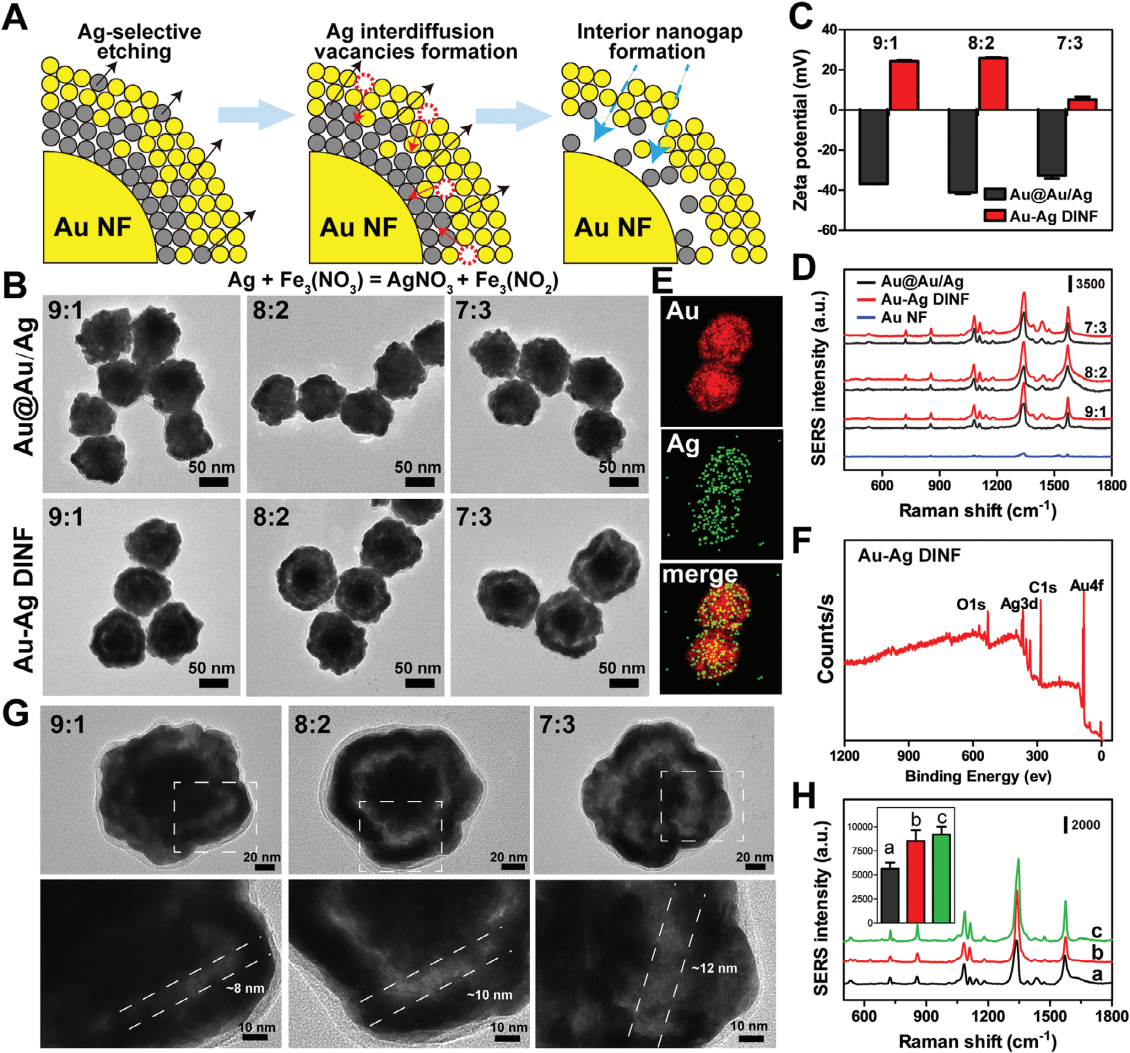
Characterization and optimization of Au–Ag DINF. A) Schematic illustration of the Ag etching process of Au–Ag DINF. B) Representative transmission electron microscopy (TEM) images, C) Zeta potential (n ≧ 10) and D) SERS spectra of Au@Au/Ag and Au–Ag DINF synthesized with different ratio of Au/Ag (9:1, 8:2, 7:3). E) The EDX elemental mapping of Au and Ag of Au–Ag DINF. F) XPS survey scan of Au–Ag DINF. G) High‐resolution TEM image of Au–Ag DINF fabricated with different ratios of Au/Ag (9:1, 8:2, 7:3). The white dotted line indicated the increase of intra‐gap size from ≈8 to ≈12 nm. H) SERS signal of Raman dye modified on the outmost shell surface (a), or the interior nanogap (b), or both outside and inside the Au–Ag‐DINF (c), respectively. Inset: Comparison of SERS intensity at I_1330_ cm^−1^. Data are presented as mean ± SD.

Afterward, selective Ag‐etching and interdiffusion of Ag atoms (SID process) happened as the introduction of etchants (Fe(NO_3_)_3_) (**Figure** **1**A). In principle, the etchant can dissolve the Ag atoms occurred on the surface of shell and diffuse inward, while Ag atoms diffused outward due to the nanometer‐scale Kirkendall effect,^[^ ^24^ ^]^ ultimately resulting in the formation of a nanometersized interior gap between the Au NF core and the alloy shell (Figure 1B). It can be seen that morphological changes from Au@Au/Ag to Au–Ag DINF induced a gradual red‐shift of absorption peak (Figure S2, Supporting Information), the transformation of zeta potential (Figure 1C), and a noticeable enhancement of SERS performance (Figure 1D). The EDX elemental maps and line scan profile sequentially demonstrated the proportion of the Ag atoms near the Au NF core decreasing during the dealloying progress (Figure 1E; Figures S4 and S5, Supporting Information). Although most of the Ag atoms in the shell region were etched away, X‐ray photoelectron spectroscopy (XPS) analysis characterized the element composition of Au–Ag DINF and revealed the existence of Au and Ag, implying that part of Ag elements still remained in the interstitial space (Figure 1F; Figure S6, Supporting Information). When the original Au NF was coated with Au shell rather than the alloy shell (Au/Ag), there was no hollow structure after the dealloying reaction (Figure S7, Supporting Information), further confirming the internal space is formed by Fe(NO_3_)_3_‐mediated Ag diffusion. It is worth noting that many approaches for preparing nanogap structures require an intermediate layer to form an intra‐nanogap, such as silica dioxide (SiO_2_)‐interlayered nanogap structures, or polymer or small molecule (DNA, amino acid)‐interlayered plasmonic nanogap structures.^[^ ^25^, ^26^, ^27^ ^]^ In this study, the selective diffusion dealloying method was used to prepare internal nanogaps without intermediate layers, which is more simple, stable, easy to control and large‐scale production. Additionally, as etchants or oxidants also undergo corrosion processes at low temperatures, this SID process can occur spontaneously at room temperature without the need for harsh reaction conditions.

It is now well‐known that interior nanogap between metallic nanostructures can significantly magnify and localize the EM field via plasmonic coupling to generate utterly strong SERS signals with very high enhancement factors. Nevertheless, the plasma coupling was greatly affected by the distance of the intra‐nanogap, which, in turn, exerted profound an influence on the amplification of SERS signal. Therefore, the optimal gap size was explored by controlling the ratio of Au/Ag shell (9:1, 8:2, 7:3), while further dealloying process was conducted as previous description. The result showed that the space of hollow interiors enlarged from ≈8 to 12 nm with the decreasing of the ratio of Au/Ag (Figure 1G; Figure S8 and S9, Supporting Information). It is reasonable because the thicker Ag layers formed, the larger the interior space formed by etching. SERS properties also increased obviously, as the abundant internal space accumulated more Raman label molecules for SERS enhancement (Figure 1D). Interestingly, irrespective of which proportion was used for fabrication, all synthesized Au–Ag DINF were uniform and monodisperse (Figure S10, Supporting Information). Additionally, the etchant to selective Ag‐etching is a paramount factor for nanogap formation, and thus, low amounts of etchant induced Ag atoms partially diffusion with an incomplete interior nanogap, while further increasing the amounts of the etchant made the internal gap clearer (Figure S11, Supporting Information). A similar trend was also noticed when the etching time was prolonged while holding etchant concentrations (20 mm) constant (Figure S12, Supporting Information). However, the SERS intensity increased with the increase of gap size, while stared to decrease as the gap size was over‐enlarged, which was primarily attributable to the weakened electric field as the gap width increased (Figures S13 and S14, Supporting Information). Together, the optimal prepared conditions were selected as 8:2 of Au/Ag ratio, 20 mm of etchants concentrations and 30 min of reaction time, in which the appropriate nanogap size can activate the optimal SERS performance of Au–Ag DINF, with the enhancement factor (EF) of 3.09 × 10^8^.

To reveal the role of intra‐nanogap on SERS enhancement further, we compared the SERS properties of Raman dye (4‐NTP) modification on the outmost shell surface or the interior nanogap of already‐formed Au–Ag DINF. As expected, since the enhanced electromagnetic field of internal nanogap and the uniform distribution of “hot spots”, its SERS signal is much higher than that attached to the outer surface of the Au–Ag DINF (Figure 1H). To further explore the optimal Raman enhancement effects, 4‐NTP was decorated at both the extra‐ and intra‐gaps of Au–Ag DINF. Notably, the SERS intensities were slightly stronger than that of the dye only anchored inside the gap. However, the dyes adsorbed on particles surface were easily disturbed by complex samples, detached from the SERS substrate, or even exchanged with another dye, often resulting in false‐positive/negative results during multi‐component PSA assay. In contrast, reporter molecules packaged inside the narrow nanogap showed long‐term SERS stability (Figure S15, Supporting Information). Considering that the Raman dye dispersed on the surface would compete with the capture antibody for binding sites, we thus adopted the strategy of fixing the Raman dye in the intra‐gap of Au–Ag DINF. It should be noted that the interior nanogap of Au–Ag DINF not only protected the Raman labels detachment, but also provided accessible “hot spots” for SERS signals stable and strong output, allowing it suitable for further highly sensitive and multiplex detection of PSA.

### 2.2 Synthesis and Characterization of AuMNPs Magnetic Capture Substrate

**Figure 2:**
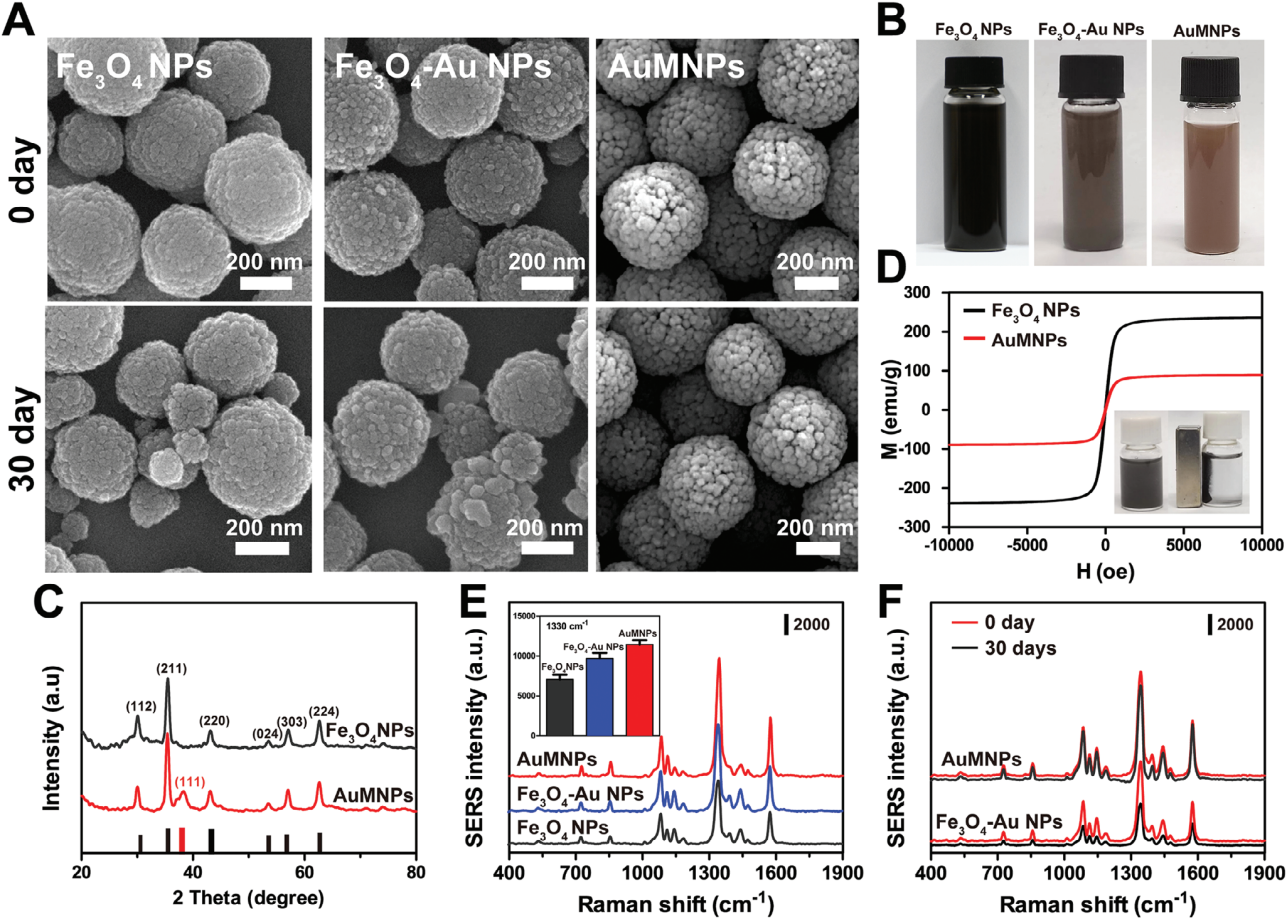
Synthesis and characterization of AuMNPs magnetic capture substrate. A) Representative TEM images of bare Fe_3_O_4_ NPs, Fe_3_O_4_‐Au NPs and AuMNPs before or after stored for 30 days. B) The color of Fe_3_O_4_ NPs, Fe_3_O_4_‐Au NPs and AuMNPs solution. C) XRD pattern and D) magnetic hysteresis of prepared Fe_3_O_4_ NPs and AuMNPs. E) SERS performance of satellite complexes triggered by all the magnetic capture substrate. Inset: Comparison of SERS intensity of (E) at I_1330_ cm^−1^ (n ≧ 6). Data are presented as mean ± SD. F) SERS performance of satellite complexes triggered by Fe_3_O_4_‐Au NPs and AuMNPs before or after stored for 30 days.

To construct magnetic capture for bio‐separation and bio‐detection of PSA, AuMNPs married magnetic and SERS response properties were fabricated in a seed‐growth method. **Figure** **2**A exhibited a scanning electron microscope (SEM) image of Fe_3_O_4_ NPs with a diameter of ≈400 nm and smooth surface, which functioned as a magnetic core for the growth of tiny Au shell. Then, Au seed (4 nm) was initially deposited on the surface of PEI‐stabilized Fe_3_O_4_ NPs to form Au‐coated Fe_3_O_4_ NPs (Fe_3_O_4_‐Au NPs), accompanying color change from black to light gray (Figure 2B). Further reduced Au atoms dwelled on the initial Au layer and grew epitaxially, eventually resulting in the formation of integral Au shell with rough surface and pinky‐grey color change (Figure 2A,B). Moreover, X‐ray diffraction (XRD) characterization of AuMNPs was displayed in Figure 2C. The diffraction peaks of Fe_3_O_4_ NPs (black curve) at 2θ values of 32.5, 35.7, 43.3, 53.7, 57.1, and 62.8 correspond to the (112), (211), (220), (024), (303), and (224) crystalline planes of the orthorhombic phase of Fe_3_O_4_ (JCPDS card no. 75–1609), respectively, which are consistent with the database of magnetite diffraction card. A new XRD pattern of AuMNPs (red curve) at a 2θ value of 38.2 was found, suggesting the (111) crystalline plane of Au (PDF 04–0784, ICDD, 2004). Consequently, XRD images further indicated the successful modification of Au shell on the outside of Fe_3_O_4_ NPs. It is worth to noting that Fe_3_O_4_ NPs were found to show slight agglomeration or aggregation during long‐stem storage due to the magnetic interaction (Figure 2A),^[^ ^28^ ^]^ while the assembly of the Au shell can shield the magnetic effect to a certain extent, allowing the AuMNPs more stable, which was also demonstrated by Magnetic hysteresis loops (Figure 2D). The magnetic saturation (MS) values of Fe_3_O_4_ and AuMNPs are 40.4 and 23.5 emu g^−1^, respectively. It is reasonable for the weak saturation strength of AuMNPs because of the shield effect of the layer of thin amorphous Au growth, as described above. But fortunately, the magnetic response of AuMNPs is still strong enough to meet the requirements of sample enrichment, captured through magnetic separation (insert in Figure 2D).

In addition to magnetic capture capability, AuMNPs also have Raman enhanced activity due to the electromagnetic enhancement of Au shell, which enabled the SERS signal of Au–Ag DINF further amplification after the formation of magnetic‐plasmonic satellite nanoassemblies. To validate the dual‐SERS enhancement effect, fPSA was used as a target, fPSA and tPSA Ab were conjugated on the surfaces of Au–Ag DINF and AuMNPs, respectively. The result showed no significant change in the characteristic SERS signal before or after Ab modification (Figure S16, Supporting Information), revealing that Ab modification would not weaken or shield the local electromagnetic field of Au–Ag DINF. The change of Zeta potential and UV–vis spectra also demonstrated the successful decoration of Ab on Au–Ag DINF (Figure S17, Supporting Information). As expected, the SERS signal of satellite nanoassemblies was significantly enhanced due to the aggregated “hot spots” between adjacent AuMNPs and Au–Ag DINF (Figure S18, Supporting Information). To further evaluate the SERS‐assisting enhancement effect of AuMNPs, we compared the SERS performance of all the magnetic capture substrate, among which SERS enhancement of AuMNPs is much stronger than bare Fe_3_O_4_ NPs and Fe_3_O_4_‐Au NPs (Figure 2E). It is possibly because the integrity of Au shell on the AuMNPs played a vital role in the enhancement of SERS signal. To further prove this hypothesis, the Fe_3_O_4_‐Au NPs and AuMNPs were stored at 4 °C for 30 days, and then formed the satellite complexes again, followed by SERS determination. The result illustrated that the SERS signal of AuMNPs still maintained its strong SERS signal since the protection effect of intact Au shell when compared with the remarkable decline SERS signal of Fe_3_O_4_‐Au NPs (Figure 2F). Considering that the dispersion of AuMNPs might affect the accuracy of detection, the SERS signal of satellite immuno‐nanocomplex was tested at different areas (*n* = 15) within the point (Figure S19, Supporting Information). The similar SERS results revealed the good reproducibility and the accuracy of our method, which benefited from the uniform morphology and dispersibility of AuMNPs (Figure S20, Supporting Information). Therefore, AuMNPs integrating the magnetic capture and SERS enhanced properties can significantly improve the sensitivity and anti‐interference of PSA assay, even in a mixed complex detection.

### 2.3 The Formation of Satellite Structure for PSA Detection

**Figure 3:**
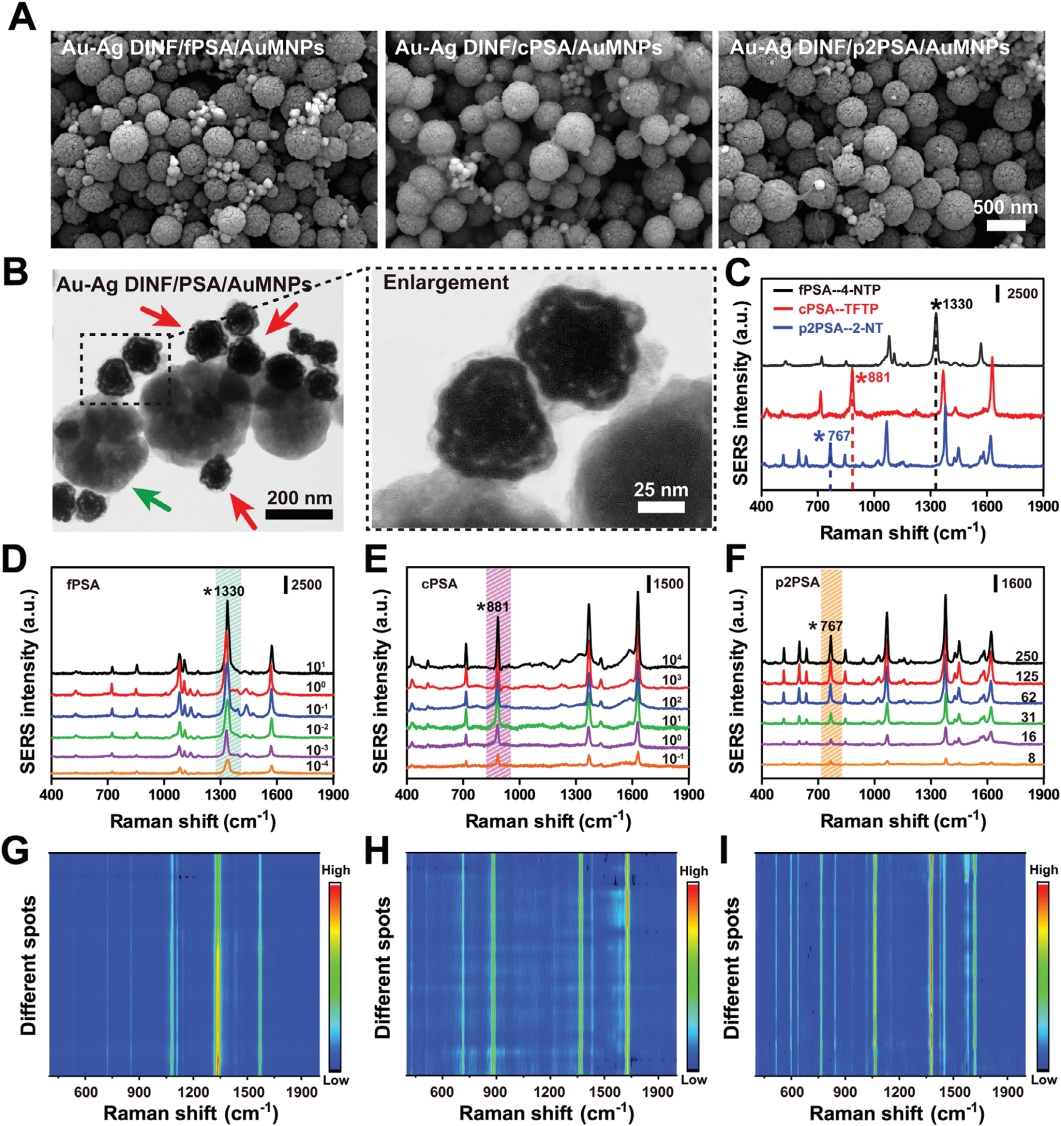
Immunocomplexes strategy for single PSA detection. A) Representative SEM images of Au–Ag DINF/fPSA, cPSA, or p2PSA/AuMNPs. B) Representative TEM and enlargement images of Au–Ag DINF/PSA/AuMNPs. The red arrows indicated the Au–Ag DINF, while the green arrows indicated the AuMNPs. C) SERS spectra of fPSA, cPSA, and p2PSA encoded with three Raman labels (4‐NTP, TFTP, 2‐NT). D–F) SERS spectra for various concentrations of fPSA, cPSA, and p2PSA in PBS. Standard curves of the SERS peak intensity at 1330, 881, and 767 cm^−1^ against the concentrations of fPSA, cPSA, and p2PSA, respectively. G,H) The corresponding 2D Raman spectra for different batches of PSA‐mediated satellite complexes (*n* = 30).

Herein, the satellite nanoassemblies were constructed based on magnetic AuMNPs (capture substrate) and Au–Ag DINF (SERS probe) for detecting different kinds of PSA. The feasibility was investigated by SERS measurement in several controlled experiments. As illustrated in Figure S21 (Supporting Information), PSA or Au–Ag DINF addition alone (group 2, 3) exhibited a negligible SERS intensities value, similar to the blank control (group 1). Furthermore, there was also no SERS signal when the AuMNPs or Au–Ag DINF without Ab decoration (group 4, 5), which inferred that bare AuMNPs or Au–Ag DINF cannot capture PSA well. Only when Ab mediated the conjugation of AuMNPs/Au–Ag DINF with PSA did the successful combination of Au–Ag DINF/PSA/AuMNPs “satellite structures” (group 6), accompanying the SERS signal emergence rapidly, suggesting the applicability of this satellite detected strategy. Moreover, SERS results were further verified by TEM images, where the Au–Ag DINF (red arrow) with obvious nanogap structure was surrounding the AuMNPs (green arrow) in the existence of PSA (**Figure** **3**B), manifesting the successful formation of the satellite nanocomplexes. To further reveal the uniform binding of Au–Ag DINF and AuMNPs, the satellite nanostructures were captured by SEM over a large range (Figure 3A). It was clear that in the presence of different target PSA, the smaller size of Au–Ag DINF were uniformly dispersed and attached to the larger size of AuMNPs, without large areas of aggregation and agglomeration. These results directly confirmed the feasibility of nanosatellite strategy, which was conducive to SERS signals uniform and reliable output.

In order to give full play to the sensing advantages of dual‐SERS satellite strategy, experiment‐concerned critical factors were optimized, respectively. For SERS analysis, the detection was performed under a 633 nm (0.5 mW) extinction laser with the strongest SERS signal, due to the proximity of the UV absorption of Au–Ag DINF (Figure S22, Supporting Information). Moreover, the maximum efficiency of the SERS probe was affected by the Ab loading. Therefore, incubated concentration and time of Ab on Au–Ag DINF was first assessed, and the results found that 4 h and 30 µg mL^−1^ of Ab was enough to obtain the satisfactory signal (Figure S23, Supporting Information). To obtain the optimal performance satellite structure, incubation time and concentration of Au–Ag DINF with AuMNPs were also measured. According to Figures S24 and S25 (Supporting Information), the optimal reaction time and concentration of Au–Ag DINF was 150 min and 250 µg mL^−1^, at which the satellite structure had the strongest SERS enhancement effect.

Subsequently, the performance of this nanocomplex strategy was evaluated in distinguishing each PSA by recording the different characteristic SERS peak of Raman label molecules. In this section, fPSA/cPSA/p2PSA Ab conjugated Au–Ag DINF (with corresponding Raman label) and tPSA Ab modified AuMNPs were respectively added into different concentrations of single component samples (fPSA/cPSA/p2PSA) to form satellite structure, followed by SERS assay. Three Raman label (4‐NTP, TFTP, 2‐NT) were selected to encode fPSA, cPSA, and p2PSA, and their corresponding characteristic peak was 1330, 881, and 767 cm^−1^, respectively, due to their no overlap and interference in the representative fingerprint identification region (Figure 3C). The optimal concentrations of 4‐NTP, TFTP, 2‐NT were 1.5, 1.2, and 1 mm, respectively, among which higher or lower concentrations were not conducive to Raman signal enhancement (Figure S26, Supporting Information). As expected, with the increase of PSA concentrations, more nanosatellite hybridization complexes were formed, resulting in the SERS intensity increased (Figure 3D–F). Figure S27 (Supporting Information) reflected an excellent linear relationship between the SERS signal (1330 cm^−1^ for 4‐NTP, 881 cm^−1^ for TFTP, and 767 cm^−1^ for 2‐NT) and the PSA concentration, with the squares of correlation coefficients (R^2^) were 0.995, 0.993, and 0.999 respectively. To further confirm the reliability of this assay, SERS spectra from different batches (30 batches for each kind of PSA) were collected and the signal variation from the corresponding representative Raman peak was observed. According to the acquired 2D SERS image (Figure 3G–I; Figure S28, Supporting Information), a uniform signal was displayed on different batches after PSA‐mediated nanosatellite formation, with a small RSD values all below 10%, implying favorable repeatability and reliability of this nanosatellite strategy.

### 2.4 The Detection of Multi‐Components PSA

**Figure 4:**
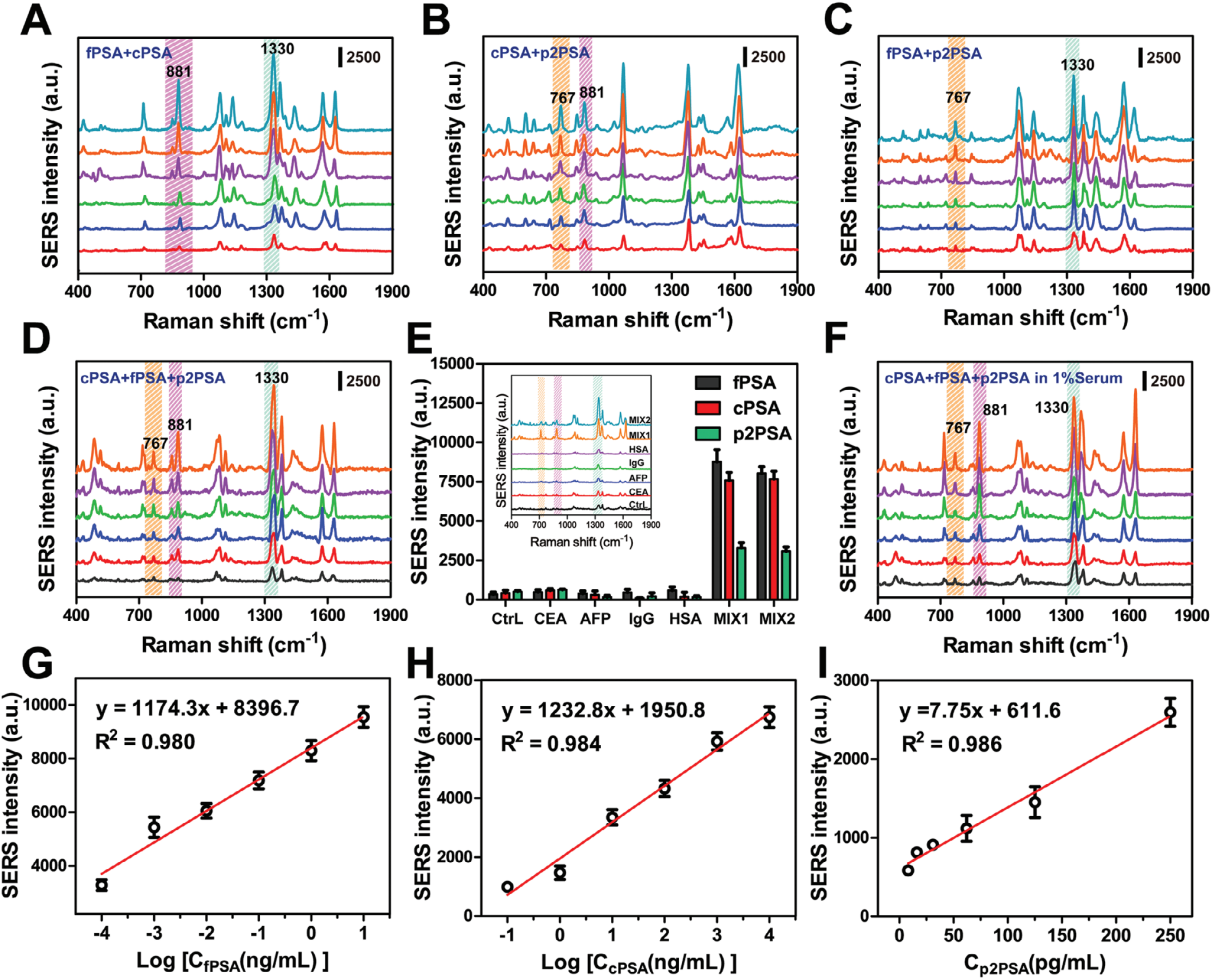
Immunocomplexes strategy for analyzing multi‐component samples. SERS spectra for simultaneous detection of A) fPSA + cPSA, B) cPSA + p2PSA, C) fPSA + p2PSA and D) fPSA + cPSA + p2PSA with different concentrations in PBS. E) Specificity evaluation of this method for interfering proteins and mixture (MIX1: fPSA+cPSA +p2PSA, MIX2: CEA+ AFP + IgG + HSA + MIX1) (n ≥ 6). Inset: SERS spectra for each group. F) SERS spectra for simultaneous detection of fPSA + cPSA + p2PSA with different concentrations in 1% serum. Standard curves of the SERS peak intensity at 1330, 881, and 767 cm^−1^ against the concentrations of G) fPSA, H) cPSA, and I) p2PSA, respectively (*n* = 6). Data are presented as mean ± SD.

To examine the effectiveness of the strategy for simultaneously analyzing multi‐component samples, double‐target PSA (**Figure** **4**A for fPSA and cPSA, Figure 4B for cPSA and p2PSA, and Figure 4C for fPSA and p2PSA) and triple‐target PSA (Figure 4D for fPSA, cPSA and p2PSA) detections were performed. Each solution mixture was incubated with specific Ab conjugated Au–Ag DINF and tPSA Ab functionalized AuMNPs, followed by two or three different types of satellite immunocomplexes formation together for SERS measurement. The corresponding multi‐characteristic SERS peaks were observed in the detection of double‐markers (Figure 4A–C). There is a linear correlation between the concentration of each PSA and corresponding SERS intensity, verifying the ability of SERS‐based strategy in double PSA biomarkers detection. Next, the performance of SERS strategy was further evaluated by concomitantly testing triple PSA samples with different concentrations. As shown in Figure 4D, SERS peak at 1330, 881, and 767 cm^−1^ showed a linear response to the concentrations of fPSA, cPSA and p2PSA over a dynamic range of 10^−4^–10^1^, 10^−1^–10^4^, and 0.008–0.25ng mL^−1^, respectively. The calibration curves were obtained for quantitative analysis of the three kinds of PSA (Figure 4G–I), the limit of detection (LOD) was calculated to be 0.11 pg mL^−1^、0.12 ng mL^−1^、0.004 ng mL^−1^ for fPSA, cPSA and p2PSA, with the R^2^ of 0.980, 0.984, and 0.986, respectively. This low LOD is comparable or even superior performance contrast of other studies for PSA determination (Table S1, Supporting Information), which was ascribed to the enhancement of EM field that was constructed by the SERS probe and capture substrate for dramatic signal amplification. Besides, PSA through magnetic separation instead of centrifugation can eliminate the interference from free Au–Ag DINF in matrices. It is noteworthy that in comparison with most previous works that mainly focus on fPSA, cPSA detection, our work also performed p2PSA detection for further PHI calculation, which would make the diagnosis of PCa more accurate and reliable. In addition, our research has a wider detection range suitable for various detected scenarios, as well as the ability to analyze multiple PSA components simultaneously.

To confirm the selectivity and specificity of this PSA‐based strategy, the SERS signal of different interfering proteins, such as CEA, AFP, IgG, and HSA, were recorded under the same conditions. A sample without any proteins served as the negative control. As given in Figure 4E, only in the presence of three PSA (MIX1) can a noticeable SERS signal be observed, while the signals of other proteins were approximately the same as that of the control group. To further unveil this strategy for specifically responding to PSA, all the interfering proteins were mixed to interfere with PSA detection (MIX2). As expected, the SERS signals obtained from MIX2 (all proteins) were nearly identical to that of the MIX1 (three target PSA). Accordingly, this SERS sensing strategy exhibited a selective response toward PSA even under interference, holding the potential in the detection of ultralow concentration PSA in complex blood samples.

Although this sensing platform showed outstanding performance for PSA detection in PBS, the high salt concentration and ionic strength in the serum might affect the stability of analysis. To reduce the severe matrix effect, fPSA as model PSA was spiked into serum samples with different dilutions. The results showed that the SERS signals obviously declined when they were applied to detect high serum specimens, while maintaining stability as the serum concentration diluted to 1%, suggesting the proposed assay can work well in 1% serum (Figure S29, Supporting Information). The UV–vis spectrum of Au–Ag DINF in 1% serum samples was similar to that in PBS buffer, further indicating good stability of assay. Next, serum samples (1%) spiked with different concentrations of three PSA samples were detected to further assess the practicability of our strategy. As shown in Figure 4F, the signal intensity on the multi‐characteristic Raman peaks increased gradually as the three spiked concentrations of PSA increased. Good recovery rates of standard addition were showed with the RSD ranging from 2.2 to 9.1% (Table S2, Supporting Information), corroborating the high availability and reliability of this strategy for real clinical sample analysis.

### 2.5 Clinical Human Samples for PSA Detection

At present, many studies mainly focus on tPSA or f/t PSA ratio test, however, insufficient specificity still cannot meet the requirement of precise detection in clinic, resulting in considerable overdiagnosis or even overtreatment of PCa patients.^[^ ^29^, ^30^, ^31^, ^32^ ^]^ There is reliable evidence that f/t PSA ratios below the cutoff were related to malignancy in only ≈42% of cases.^[^ ^33^ ^]^ As a result, for patients with PSA values in the “gray area”, conducting biopsies based on the f/t PSA ratio is like throwing a coin. According to preliminary investigations and observational studies, PHI, approved by the FDA in 2012,^[^ ^34^ ^]^ effectively decreases the number of biopsies that could be avoided and the number and pathologic characteristics of cancers that would be missed. PHI is a new formula that combined all three forms (tPSA, fPSA, and p2PSA) into a single score, implying an increased positive predictive value when compared with tPSA or f/t PSA ratio. Herein, different from the previous studies, this study adopted multiplexed PSA immuno‐SERS sensing and integrated them with PHI strategy toward PCa screening, with the aim to further minimize the need for biopsies.

To determine the discriminative capability of the proposed strategy in clinic, an in‐depth study of 23 serum samples, including 5 healthy individuals (H1‐H5, tPSA<4 ng mL^−1^), 10 PCa suspicious patients (P6‐P15, tPSA in 4–10 ng mL^−1^) and 8 PCa positive patients (P16‐P23, tPSA>10 ng mL^−1^) were conducted under identical conditions. Fresh tissues from 23 patients were sent to the pathology department for standard examination. The multiple PSA levels were measured simultaneously and converted from the standard curves of SERS signal, while f/t PSA% and PHI [p2PSA/fPSA×√(tPSA)] were calculated as the index tests. The result of PHI obtained by SERS system was not used as the basis for any medical behavior and did not interfere with routine medical procedures.

**Figure 5:**
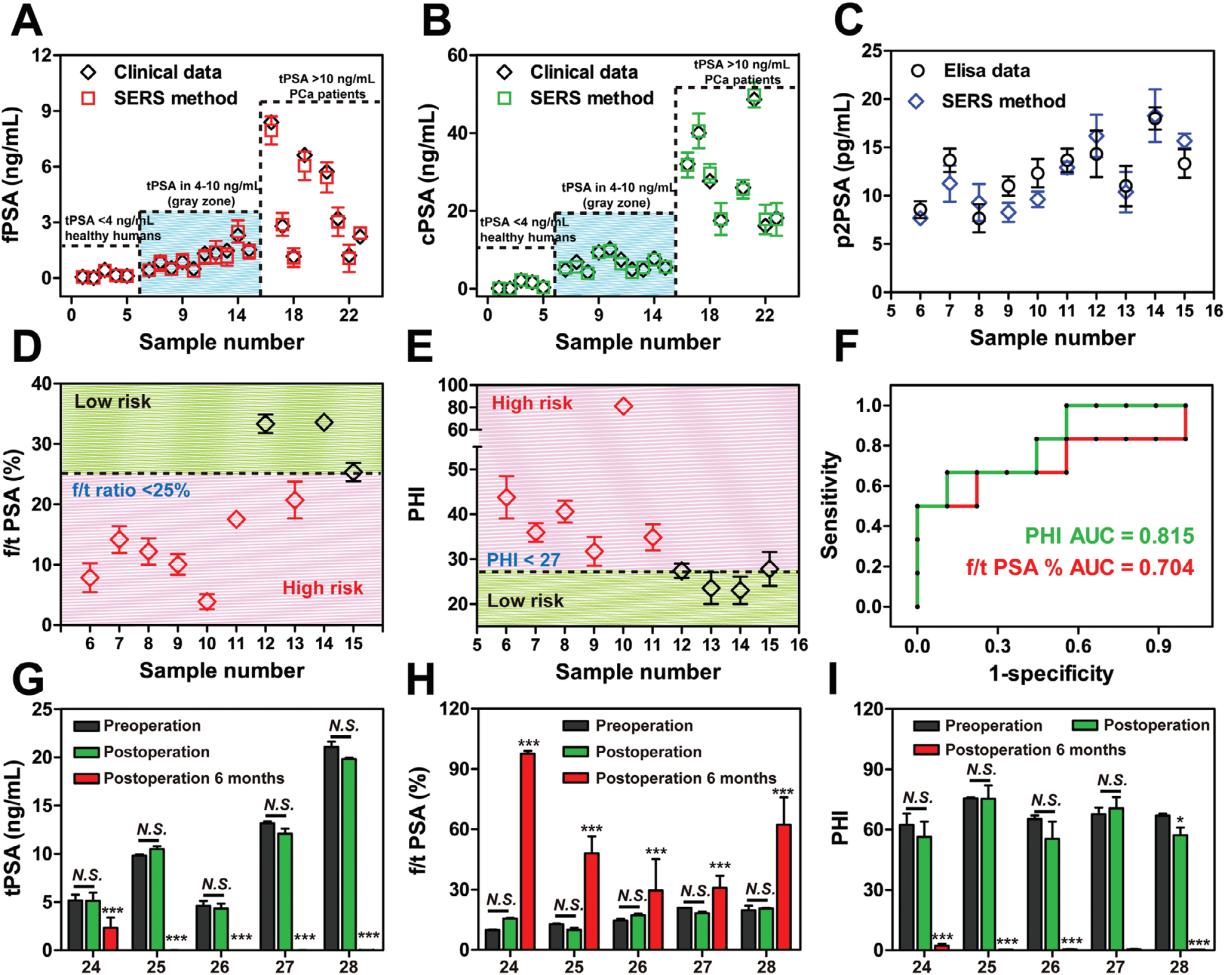
Clinical application of immunocomplexes strategy in human serum samples. A) fPSA levels and B) cPSA levels of healthy humans (*N* = 5, healthy_1‐5_), PCa suspicious patients (*N* = 10, patients_6‐15_) and PCa positive patients (*N* = 8, patients_16‐23_). All samples were classified according to their tPSA values. The black tag represented the clinical test results, while the red/green tag indicated the SERS analysis results. C) p2PSA levels of PCa suspicious patients (*N* = 10, patients_6‐15_), whose tPSA values were in the gray zone. The black tag represented the ELISA test results, while the blue tag indicated the SERS analysis results. D) f/t PSA% of PCa suspicious patients (*N* = 10, patients_6‐15_), where 25% was set as the cutoff value for evaluation. E) PHI of PCa suspicious patients (*N* = 10, patients_6‐15_), where 27 was set as cutoff values for consideration. F) ROC curves of classification results for PCa patient based on f/t PSA% and PHI. G) tPSA levels, H) f/t PSA% and PHI obtained from patients (*N* = 5, patients_24‐28_) before, after operation or 6 months after operation. *p < *0.05*, ***p < *0.001*, *N.S. = No significance*, Data are presented as mean ± SD and tested at least in triplicate (n ≧ 3).

A comprehensive comparison between the proposed method and clinical bioassay technologies for these 23 clinical samples was shown in **Figure** **5**A,B, where the fPSA and cPSA results were concordance very well with that of the hospital data, further demonstrating the effectiveness of our SERS method for simultaneous detection of multiple PSA in clinical samples. It is notable that fPSA levels in PCa patients (P16‐P23) measured by hospital or our method did not decrease, which illustrated that fPSA alone is not enough to accurately distinguish PCa. Fortunately, all subjects with a tPSA<4 ng mL^−1^ showed PCa negative, while all subjects with a tPSA>10 ng mL^−1^ were diagnosed as PCa positive. However, tPSA values in the “diagnostic gray zone” have been a key issue limiting the predictive efficiency of PSA in PCa, as described in the Introduction Section. We therefore focused on 10 PCa suspected patients (P6‐P15) whose tPSA values were in the gray zone. It was previously reported that f/t PSA% less than 25% indicated the high risk of harboring PCa.^[^ ^35^, ^36^ ^]^ Therefore, the f/t PSA% was further calculated according to single PSA level as detected above, and the results depicted that seven samples (P6‐P11 and P13) had abnormal cutoff values of <25% f/t PSA ratio, while it still needs to further evaluation to make an accurate judgment (Figure 5D).

Since PHI can be used as a reflex test when f/t PSA% is 25% or less, PHI was introduced to further improve the diagnostic accuracy for PCa. However, the hospital only provided the level of fPSA and tPSA without p2PSA, whereas the simultaneous detection of p2PSA can be achieved in our study benefiting from the satellite immunocomplexes strategy. As shown in Figure 5C, the p2PSA value of P6‐P15 samples ranged from 5 to 20 pg mL^−1^, which was consistent with the p2PSA ELISA kit, further confirming the accuracy and reliability of our assay. For PHI analysis, the data set recommended by the CSCO‐PC Diagnostic Guide suggested that the likelihood of a positive biopsy is 9.4% if the PHI is less than 27, while the likelihood increased to 66.4% if the PHI is more than 55. Therefore, we set the cutoff values of PHI at 27, from which the PCa subjects were effectively discriminated from normal ones. It can be seen that all patients identified as high risk by f/t PSA% (P6‐P11) were consistent with PHI assessment, except for P13, whose low PHI indicated a relatively low risk of harboring PCa (Figure 5E). The resected specimen was subsequently examined and, as predicted by PHI, P13 was diagnosed as BPH or prostatitis rather than PCa (Figure S30, Supporting Information). To further reveal the reliability of PHI method, we observed the pathology section of positive sample P6, and compared it with P13. Unlike P13, whose tissue was uniform with only partial inflammatory cell infiltration, P6 patients have obvious malignant lesions (Figure S31, Supporting Information), where the shape and arrangement of tumor cells were obviously different from normal tissues, and the cell nuclei of cancer cells were large and irregular. What this indicates is that this new PHI test outperforms its individual components of tPSA, fPSA and f/t PSA% in PCa prediction, which was also supported by many studies.^[^ ^37^, ^38^, ^39^ ^]^

Next, predicted discrimination of the different models was visualized using receiver operating characteristics (ROC) curves, which are conducive to differentiating false‐positive and false‐negative results in different clinical scenarios. In the analysis of all patients, PHI (0.815) displayed a robustly greater AUC compared with f/t PSA% (0.704), indicating the satisfactory performance of PHI model for identifying PCa (Figure 5F). Even so, we suggested that integration of multiple combined test (tPSA, f/t PSA% and PHI) rather than a one‐size‐fits‐all approach would provide more valuable information for prostatologist in PCa surveillance.

Observing the prognosis of patients is of paramount importance to tracing the effect of treatment. Furthermore, we considered whether these markers/models could provide guidance for the prognosis of PCa patients, which contributed to tracking the progression of the disease in clinic. The changes in PSA level (tPSA, f/t PSA% and PHI) were monitored on 5 PCa patients (P24‐P28) before and after surgery. The results in Figure 5G–I depict that there was no significant difference in tPSA level before and after surgery even though most of PCa lesion was removed by surgery, probably because of the slow regulation of tPSA in the blood circulation. However, some important differences appeared six months after their operations, where tPSA level and PHI sharply fell off while f/t PSA% significantly increased, indicating a gradual recovery of the PCa patients. The information of PCa clinical samples and original data, including the tPSA level and f/t PSA% measured according to our method and clinical assay, are recorded in Table S3 (Supporting Information). All the above encouraging results toward an application demonstrated the availability of this SERS‐based PHI method for early diagnosis and prognosis guidance of PCa, especially for clinical samples in the “gray zone”. Regrettably, patients included in the study were based on their risk of carrying PCa and not for general screening purposes, and therefore, the results of the current study may be limited by this selectivity bias, as all samples were selected according to their tPSA values.

## 3 Conclusion

In summary, we herein developed an Au–Ag DINF with robust, stable, and reproducible SERS signals and integrated them with AuMNPs magnetic capture substrate to form satellite immunoassemblies toward highly sensitive PSA‐specific sensing. Unlike the conventional studies that mainly focused on tPSA and f/t PSA%, this work introduced a multiple PSA‐mediated PHI assessment for improving the accuracy and specificity of PCa diagnosis, which is expected to provide the increased predictive value of PSA. This proposed method exhibited a good linear relationship of each PSA with low LOD in 0.11 pg mL^−1^, 0.12 ng mL^−1^, and 0.004 ng mL^−1^ for fPSA, cPSA and p2PSA, respectively. In clinical application, we demonstrated that the multiple PSA levels of 23 samples determined by proposed assay corresponded well with clinical reference values. Based on these favorable performances, our assay could successfully identify PCa subjects with the assistance of f/t PSA% and PHI tests, particularly the patients who are in the “diagnostic gray zone”. Through tracing the PSA level of 5 PCa patients before and after surgery, this study offered the possibilities for monitoring prognosis of PCa. Future work will further immobilize the SERS probe and capture substrate on one chip to simultaneously sensing multiple PSA, with the aim of achieving high throughput and rapid detection. Finally, the combination of PHI calculation with mobile apps on smartphones and tablets would make it become an ideal point‐of‐care device for more convenient use in clinical settings.

## 4 Experimental Section

### Materials and Reagents

Hydrogen tetrachloroaurate (HAuCl_4_⋅4H_2_O), polyvinylpyrrolidone (PVP), silver nitrate (AgNO_3_), lascorbic acid (AA), trisodium citrate, N‐(3‐dimethylaminopropyl)‐N’‐ethylcarbodiimide hydrochloride (EDC), N‐hydroxysuccinimide (NHS), TFTP, 2‐NT, 4‐NTP and phosphate‐buffered saline (PBS) were obtained from Macklin (Shanghai, China). Iron (III) nitrate nonahydrate (Fe(NO3)3·9H2O), ammonium hydroxide solution (NH4•OH), and hydroquinone were purchased from Aladdin (Shanghai, China). Sodium borohydride (NaBH_4_) was obtained from Sigma Co., Ltd. (St. Louis, USA). Monoclonal fPSA antibody (Ab) was supplied from Shanghai Linc‐Bio Science Co., Ltd (China). Monoclonal cPSA Ab was purchased by Biospacific (USA). Polyclonal p2PSA Ab was purchased by Jiangsu Meimian Co., Ltd (China). The water (H_2_O) used in this study was purified via an ultrafilter system (Milli‐Q, Millipore, Marlborough, MA). The blood samples from health human or PCa patients were supplied by the Sun Yat‐sen University Cancer Center (Guangzhou, China).

### Synthesis of Au–Ag DINF

Au NPs was fabricated in a trisodium citrate reduction method. At first, 0.25 mL of HAuCl_4_ solution (0.1 m) was added into 100 mL of ultrapure water, and heated to boil under magnetic stirring. Then, 1.5 mL of sodium citrate solution (1%) was rapidly added once the solution was boiled, and reacted for 30 min. During this process, the color of the solution changed from colorless to black and finally to red‐purple, indicating that the reaction was complete. After that, the Au colloidal suspension was stored in foil‐wrapped glass bottles at 4 °C for further use.

Au NF was prepared by referring a previously reported procedure with minor modifications.^[^ ^40^ ^]^ Typically, 1.78 mL as‐prepared Au NPs (≈20 nm), 1.2 mL 1% HAuCl_4_ and 2.64 mL 1% trisodium citrate were dispersed into the 100 mL aqueous solution under gentle stirring. After heating to 50 °C, 24 mL hydroquinone (30 mm) was added to the above solution with vigorous stirring, accompanying color change to blue‐grey. Finally, the resulting Au NF was centrifuged (6000 rpm, 10 min) to remove residue, and washed with ultrapure water three times.

For synthesis of Raman reporter‐modified Au NF, 1 mL of Au NF was mixed with 25 µL of different Raman labels (1.5 mm of 4‐NTP, 1.2 mM of TFTP, 1 mM of 2‐NT) and incubated for 24 h at 220 rpm and 25 °C. The mixture was centrifuged to remove free Raman label molecules at 6000 rpm for 10 min, and then redispersed with ultrapure water, the labeled Au NF was obtained and stored at 4 °C for further use.

For the preparation of Au@Au/Ag, the obtained 200 µL Au NF was added into 200 µL 1% PVP and stirred gently. Then, 120 µL AgNO3 solution (1 mm), 16 µL NH4•OH solution, and 280 µL HAuCl4 solution (1 mm) were sequentially added to the mixture. Afterward, 200 µL AA solution (20 mm) was immediately injected into the mixture and incubated at room temperature for 1 h in a dark place. The mixture was centrifuged at 6000 rpm for 10 min, and then redispersed with ultrapure water, the Au@Au/Ag were obtained and stored at 4 °C for further use. In this experiment, Au@Au/Ag with different Au/Ag shell thickness (9:1, 8:2 and 7:3) were prepared by changing the volume of added HAuCl4 and AgNO3 solution (360/40, 320/80, and 280/120 µL).

For the preparation of Au–Ag DINF, the obtained 100 µL Au@Au/Ag was mixed with 100 µL 1% PVP under gently stirring. Then, 125 µL Fe(NO3)3 was injected slowly into the mixture with stirring for 30 min at room temperature. The resulting Au–Ag DINF was washed with distilled water by centrifugation at 8000 rpm for 10 min and redispersed in distilled water. To specifically capture fPSA/cPSA/p2PSA, Au–Ag DINF were activated by incubation with 5 µL EDC (0.1 m) and 5 µL NHS (0.1 m), followed by a reaction with corresponding fPSA/cPSA/p2PSA Ab for 4 h with continuous shaking as previous studies.^[^ ^41^, ^42^, ^43^ ^]^ In this experiment, the optimal etching ratio of Au–Ag DINF was explored by varying the concentration of Fe(NO3)3 (5, 10, 20, 30, 40 and 50 mm); the optimal etching time of Au–Ag DINF was explored by varying the incubation time of Fe(NO3)3 (10, 20, 30, 40, and 50 min).

### Calculation of Enhancement Factor

For the calculation of enhancement factor (EF), the well‐established equation was introduced as follows:

(1) $$EF=\frac{I_{\mathrm{SERS}}\times N_{\mathrm{bulk}}}{I_{\mathrm{bulk}}\times N_{\mathrm{SERS}}}$$

*I*_bulk_ and *I*_SERS_ represent the intensity of analyte in solution for bulk Raman spectra and SERS, respectively. *N*_bulk_ and *N*_SERS_ are the number of molecules in the spots excited by the laser beam in bulk Raman spectra and SERS, respectively.

(2) $$N_{\mathrm{SERS}}=N_{A}\times CV\frac{S_{\mathrm{Laser}}}{S_{\mathrm{sub}}}$$

*N_A_* indicates Avogadro constant; C and V correspond to the molar concentration and volume, respectively; *S*_Laser_ and *S*_Sub_ are the size of the laser spot and substrate, respectively.

(3) $$N_{bulk}=N_{A}\times P_{v}\;S_{Laser}$$

*Pv* [mol µm^−3^] represents the volume density of 4‐NTP powder on a glass slide. During this calculation process, mass density of 4‐NTP powder is 1.362 g cm^−3^, while molecular weight of 4‐NTP is 155 g mol^−1^, thus it can be calculated as *Pv* [mol µm^−3^] = (1.362/155) × 10^−12^ = 8.79 ×10^−15^ mol µm^−3^.

(4) $$EF=\frac{I_{SERS}\times P_{v}\;S_{Laser}}{I_{bulk}\times CV}$$

In this experiment, 25 µL of 4‐NTP (1.5×10^−9^ M) was mixed with Au–Ag DINF, which was then dropped on a glass slide and dried in the air to form a circle with a diameter of 5362 µm. The Raman signal of 4‐NTP was analyzed at the 1330 cm^−1^ characteristic peak, where 12 601.8 counts of *I_SERS_* was significantly stronger than 862.0 counts of *I_bulk_*. The EF can be calculated as: EF = (12 601.8 counts×8.79×10^−15^ mol µm^−3^×(5362 µm)^2^×3.14)/(862.0 counts×1.5×10^−9^ M×25 µL×10^−6^) = 3.09×10^8^.

### Synthesis of AuMNPs

At first, the Fe_3_O_4_ was synthesized by referring to the previous report with little modification.^[^ ^44^ ^]^ In brief, 2 g FeCl_3_⋅6H_2_O was dissolved in 60 mL of ethylene glycol followed by the addition of 1.5 g PEG (Mw = 10 000) and 3.25 g of NaAc⋅3H_2_O. After stirring overnight, the mixture was transferred to Teflonlined stainless‐steel autoclave, and heated to 200 °C for 8 h. The black precipitate was collected, magnetically rinsed with ethanol and ultrapure water five times, and dried at 60 °C.

To assemble AuNPs on the surface of Fe_3_O_4_ (Fe_3_O_4_‐Au NPs), PEI was utilized as a binding agent via electrostatic interaction. 0.25 g PEI was dissolved in the 50 mL of ultrapure water, and then added into Fe_3_O_4_ solution under sonication for 20 min. The resulting Fe_3_O_4_@PEI NPs were rinsed five times with ultrapure water by magnetic separation. Au seed (4 nm) was synthesized following the previous method with some modification.^[^ ^45^ ^]^ Then, 120 mL Au seed (4 nm) was mixed with 20 mL Fe_3_O_4_@PEI NPs and sonicated for 1 h. The as‐fabricated Fe_3_O_4_‐Au NPs was magnetically washed three times with water and ethanol and then dried at room temperature.

For further synthesis of AuMNPs, 0.3 mL HAuCl_4_ (0.05 M) was added into the obtained Fe_3_O_4_‐Au NPs; subsequently, sonicated for 5 min under 30 °C. Then, 100 mg NH_2_OH⋅HCl was added to the solution and mixed by sonication, followed by adding 600 mg PVP under sonication for another 30 min. Finally, the resulting Au MNPs were magnetically washed three times with water and stored for future use. To specifically capture tPSA, AuMNPs were activated by incubation with 5 µL EDC (0.1 m) and 5 µL NHS (0.1 m), followed by reacting with 15 µL anti‐tPSA Ab (1 mg mL^−1^) for 4 h with continuous shaking, leading to the conjugation of tPSA Ab on the surface of AuMNPs.

### PSA detection from PBS media

Typically, the fPSA/cPSA/p2PSA was diluted with PBS to the desired concentrations. First, 200 µL fPSA/cPSA/p2PSA Ab decorated Au–Ag DINF were incubated with 200 µL target fPSA/cPSA/p2PSA solution of different concentrations at room temperature for 150 min. Subsequently, 200 µL tPSA Ab modified AuMNPs were added and incubated with the mixture at room temperature for another 2 h. Then, the Au–Ag DIN/PSA/AuMNPs nanocomplexes were obtained through magnetic separation and washed with PBS to remove unbound PSA. For two components, and multi‐components detection, two (three)‐Ab modified Au–Ag DINF, were added and incubated with complexes, while other approach was the same as that of single component detection. For SERS assay, the Raman spectra were measured by a Raman microscope (LabRAM HR, Horiba, Ltd., Kyoto, Japan) with a 2 s integration time and 2 accumulations. The parameters of measurement are set as follows: 633 nm laser, 50 × telephoto objective at a laser power of 0.5 mW. To ensure the detection reliable, at least 10 spectra were collected at different locations within the point region for each sample and analyzed on average. Data acquisition and processing were conducted by the LabSpec software, and all Raman spectra were presented through baseline adjustment.

To evaluate the specificity of assay, other several interferents, namely, CEA, AFP, IgG, and HSA were also measured as target PSA detection under the same experimental conditions. To determine the stability and repeatability, the above Au–Ag DINF were stored in the dark at 4 °C for different days and then conducted to PSA detection by SERS sensing. To increase the statistics, at least 30 different points of each group were collected, and the average value was taken as the final result. The LOD was calculated according to the ratio of the standard deviation of the blank to the slope of the linear equation of the calibration plot (LOD = 3 SD/slope, *n* = 3).^[^ ^46^ ^]^

### Assay of Clinical Serum Samples

To determine the clinical applicability and diagnostic capability of the proposed strategy, a total of 28 clinical serum samples provided by the Sun Yat‐sen University Cancer Center (Guangzhou, China) were conducted to the assays. The study was conducted in accordance with clinical guidelines and approved by the local Bio‐Ethics Committee. All samples were obtained from individual patients with written informed consent (B2024‐794‐01). Given the high salt concentration and ionic strength in the serum may affect the stability of analysis, the serum was diluted to 1% with PBS for all of the following experiments. The SERS signals of fPSA, cPSA, and p2PSA were detected using the SERS‐based assay and the concentration were calculated according to the standard curves. The ratios of f/t PSA and PHI were also calculated from the detected concentration of PSA. Finally, the SERS‐based strategy results were compared with those measured by the hospital assay or ELISA assay.

To confirm the accuracy of the SERS method, the ELISA kit was used to determine the p2PSA level in the specimen based on the double antibody sandwich assay. The p2PSA antibody was first coated with microporous plate, and pure p2PSA was added into the micropores coated with antibody successively, and then combined with HRP labeled p2PSA antibody to form antibody‐antigen‐enzyme‐labeled antibody complex. After thorough washing, TMB substrate was added for colorimetric observation. Finally, the concentration of p2PSA in the sample was measured at 450 nm absorbance, and calculated by standard curve. It was notable that the detected range of this ELISA kit was 6–280 pg mL^−1^.

### Statistical Analysis

Data were analyzed and graphed using GraphPad Prism 9.0 (GraphPad Software Inc.). All experiments were carried out at least in triplicate (n ≧ 3) and all data presented as mean ± S.D. Statistical significance was assessed using ANOVA analysis and by independent t‐tests for pairwise comparisons. p‐value of <0.05 was regarded as statistically significant, *N.S*. was regarded as no significant difference (*N.S*. = no significant difference; *p < *0.05*).

### Ethics Approval Statement

This protocol was developed to be consistent with the ethical principle described in the local Bio‐Ethics Committee and received consent from the Ethical Review Committees of Sun Yat‐sen University Cancer Center (Guangzhou, China).

### Patient Consent Statement

All samples were obtained from individual patients with written informed consent (B2024‐794‐01).

## Conflict of Interest

The authors declare no conflict of interest.

## Author Contributions

D.C., Y.M., and A.Y. contributed equally to this work. D.C. performed conceptualization and project administration. Y.M. performed conceptualization, data curation, and formal analysis. A.Y. performed investigation and methodology. L.H. performed investigation and methodology. H.Z. performed investigation and methodology. J.X. performed conceptualization and formal analysis. S.C. performed formal analysis and investigation. D.N. performed formal analysis and investigation. W.F. performed formal analysis and investigation. H.C. performed investigation and methodology. Y.C. performed investigation and methodology. J.P. performed methodology, acquired resources, and software. L.R. acquired resources, software, and performed supervision. X.H. performed conceptualization, data curation, funding acquisition, writing. P.S. performed project administration and supervision. H.Z. performed project administration, supervision, and writing.

## Supporting information



Supporting Information

## Data Availability

The data that support the findings of this study are available from the corresponding author upon reasonable request.
