# Supplementary material for: Dual‐Enhanced SERS Satellite Immuno‐Nanocomplex for Multiple PSA‐Mediated PHI Assay Toward Clinical Prostate Cancer Screening
Source: Adv Sci (Weinh). 2024 Dec 10;12(5):2411747. doi: 10.1002/advs.202411747 (PMC11791982; doi:10.1002/advs.202411747)
Supplement: Supplementary file 1 — Supporting Information [file ADVS-12-2411747-s001.pdf]

ADVANCED SCIENCE
Open Access

Supporting Information

for *Adv. Sci.*, DOI 10.1002/advs.202411747

Dual-Enhanced SERS Satellite Immuno-Nanocomplex for Multiple PSA-Mediated PHI Assay Toward Clinical Prostate Cancer Screening

Dong Chen, Yilin Ma, Annan Yang, Liping Hu, Hanlin Zhou, Jun Xu, Shanze Chen, Dingmeng Nie, Weifeng Feng, Huaihong Cai, Yanguang Cong, Jiang Pi, Lang Rao, Xueqin Huang*, Pinghua Sun* and Haibo Zhou**

Supporting Information

Dual-Enhanced SERS Satellite Immuno-Nanocomplex for Multiple PSA-Mediated PHI Assay toward Clinical Prostate Cancer Screening

Dong Chen [1, 2], Yilin Ma [1, 3], Annan Yang [1, 3], Liping Hu [1, 3], Hanlin Zhou [1, 3], Jun Xu [1, 3], Shanze Chen [1, 3], Dingmeng Nie [4], Weifeng Feng [5], Huaihong Cai [6], Yanguang Cong [7], Jiang Pi [7], Lang Rao [4, *], Xueqin Huang [7, *], Pinghua Sun [1, 3, *], Haibo Zhou [1, 3, *]

[1] Institute for Safflower Industry Research, Key Laboratory of Xinjiang Phytomedicine Resource and Utilization (Ministry of Education), School of Pharmacy, Shihezi University, Shihezi 832003, China.

[2] Department of Urology, State Key Laboratory of Oncology in South China, Sun Yat-sen University Cancer Center, Guangzhou 510060, China.

[3] College of Pharmacy, The Second Clinical Medical College (Shenzhen People's Hospital), The Fifth Affiliated Hospital, Jinan University, Guangzhou 510632, China.

[4] Institute of Chemical Biology, Shenzhen Bay Laboratory, Shenzhen 518132, China.

[5] The First Affiliated Hospital of Jinan University, Guangzhou 510632, China.

[6] College of Chemistry and Materials Science, Jinan University, Guangzhou 510632, China

[7] Guangdong Provincial Key Laboratory of Medical Molecular Diagnostics, The First Dongguan Affiliated Hospital, School of Medical Technology, Guangdong Medical University, Dongguan 523000, China.

D.C., Y.M., and A.Y. contributed equally to this work.

* Corresponding authors:

lrao@szbl.ac.cn (Lang Rao), xqhuang@jnu.edu.cn (Xueqin Huang), Sunph@jnu.edu.cn (Pinghua Sun), and haibo.zhou@jnu.edu.cn (Haibo Zhou)

Table of Contents

Figure S1 4
Figure S2 4
Figure S3 4
Figure S4 5
Figure S5 5
Figure S6 5
Figure S7 6
Figure S8 6
Figure S9 7
Figure S10 7
Figure S11 8
Figure S12 8
Figure S13 9
Figure S14 9
Figure S15 10
Figure S16 10
Figure S17 10
Figure S18 11
Figure S19 11
Figure S20 12
Figure S21 12
Figure S22 13
Figure S23 13
Figure S24 14
Figure S25 14
Figure S26 14
Figure S27 15

Figure S28 15

Figure S29 16

Figure S30 16

Figure S31 17

Table S1 18

Table S2 18

Table S3 19

Supplementary Figures and Table

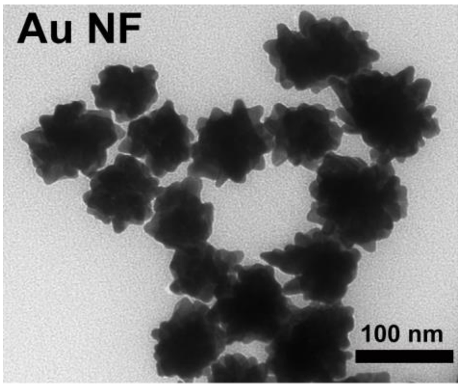

Figure S1. TEM images of Au NF.

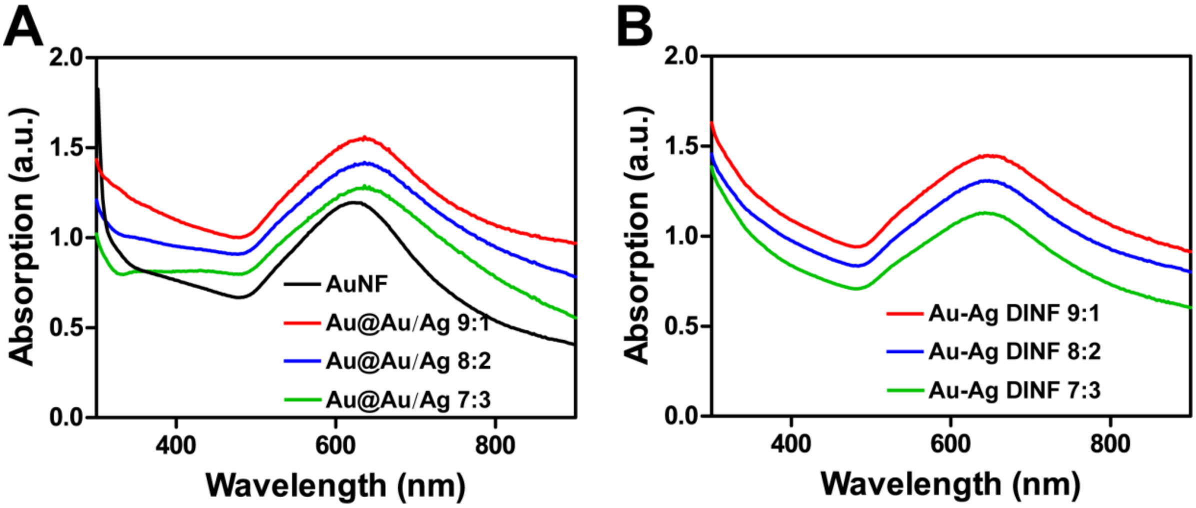

Figure S2. (A) UV-vis absorption spectra of Au NF and Au@Au/Ag synthesized with different ratio of Au/Ag (9:1, 8:2, 7:3). (B) UV-vis absorption spectra of Au-Ag DINF synthesized with different ratio of Au/Ag (9:1, 8:2, 7:3).

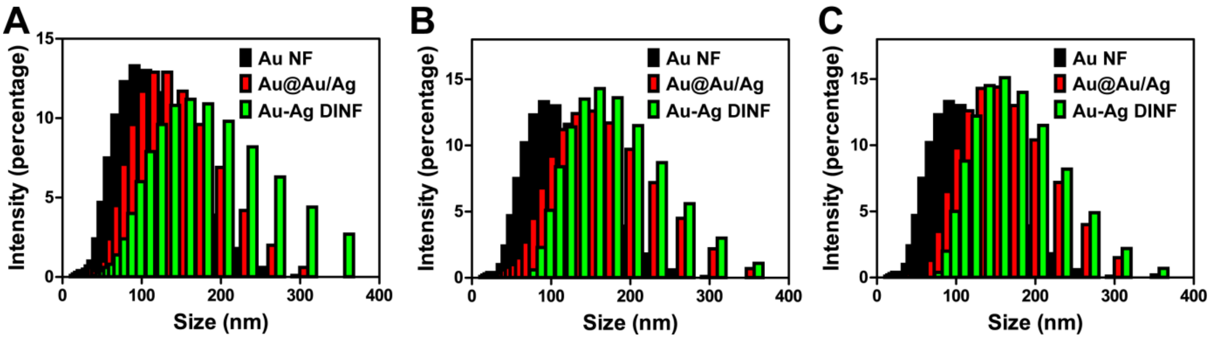

Figure S3. (A-C) Hydrodynamic diameter of Au NF, Au@Au/Ag and Au-Ag DINF synthesized with different ratio of Au/Ag (A for 9:1, B for 8:2, C for 7:3).

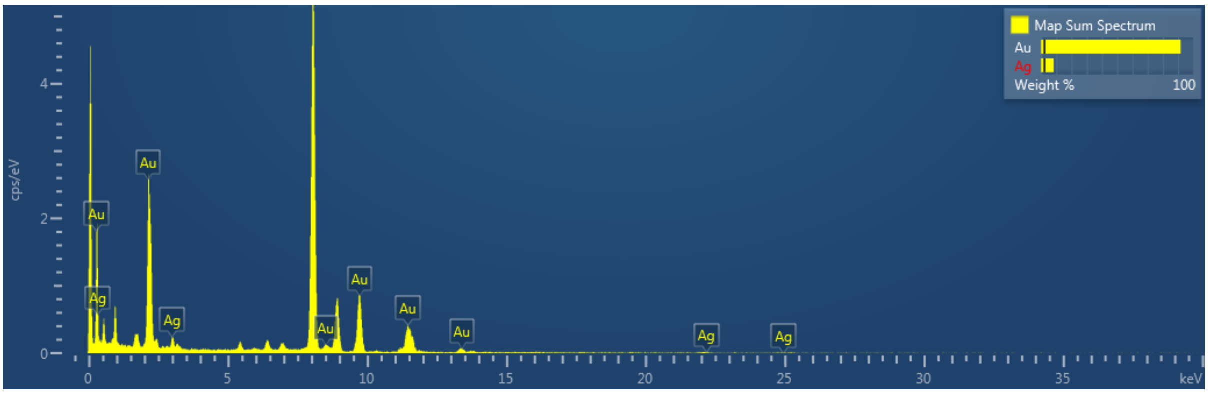

Figure S4. TEM-EDS spectrum of Au-Ag DINF.

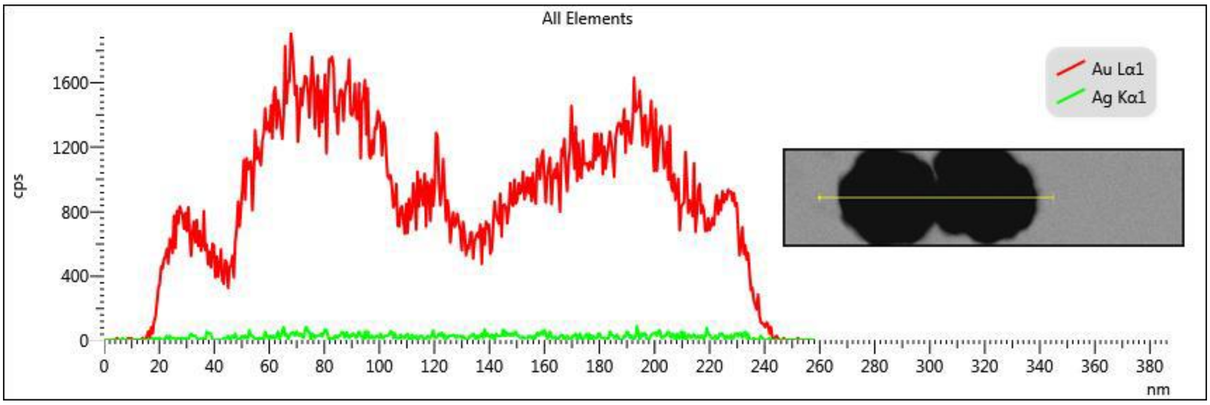

Figure S5. EDX line scan of Au-Ag DINF.

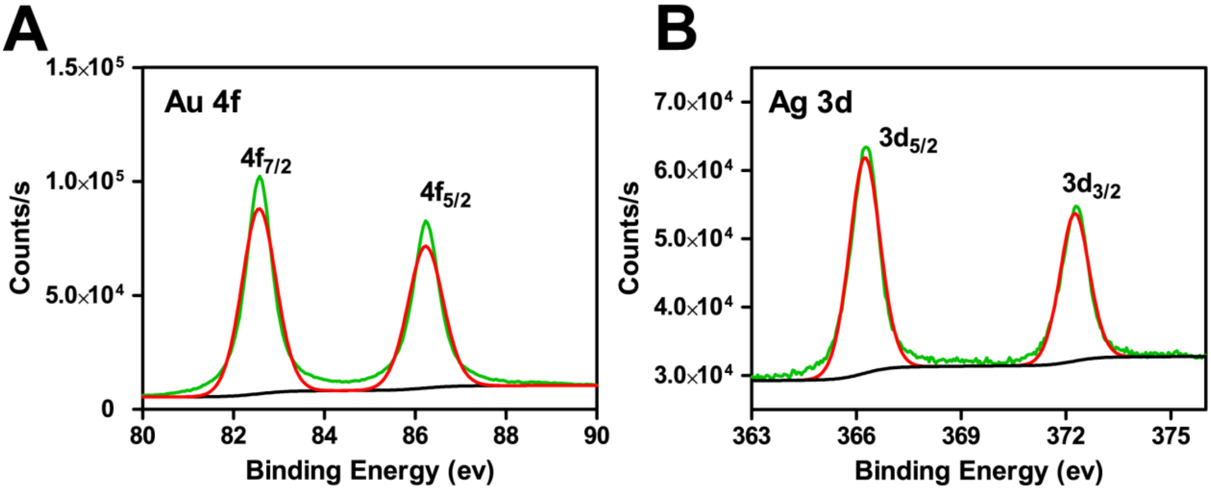

Figure S6. (A) High-resolution Au 4f and (B) Ag 3d XPS spectra of Au-Ag DINF.

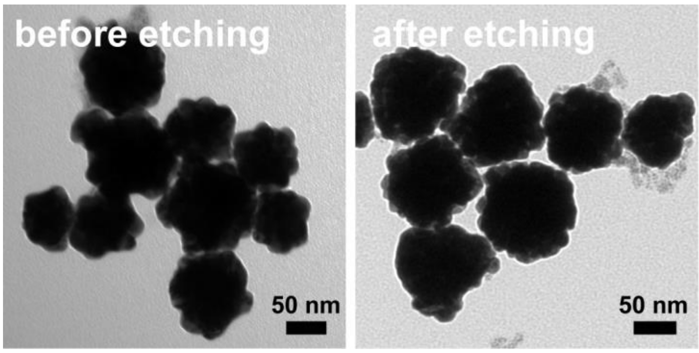

Figure S7. Representative TEM images of Au NF coated with Au shell before or after etching.

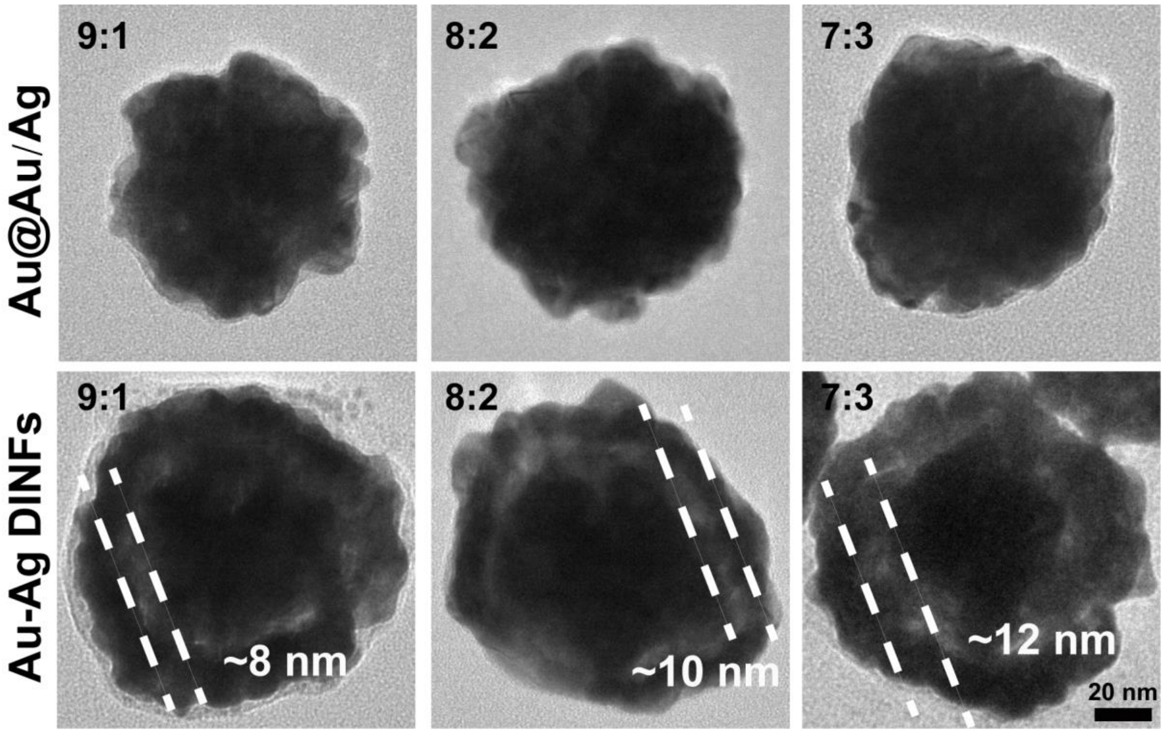

Figure S8. High-resolution TEM image of Au@Au/Ag and Au-Ag DINF fabricated with different ratio of Au/Ag (9:1, 8:2, 7:3). The white dotted line indicated the increased interior nanogaps of Au-Ag DINF.

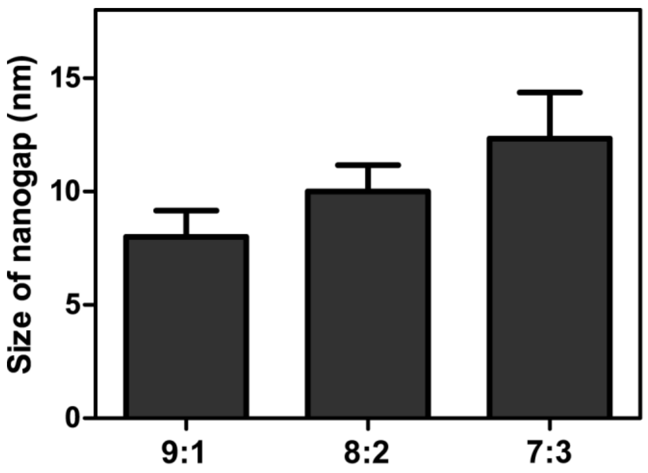

Figure S9. The intra-gap size of Au-Ag DINF (9:1, 8:2, 7:3) measured by the high-resolution TEM images.

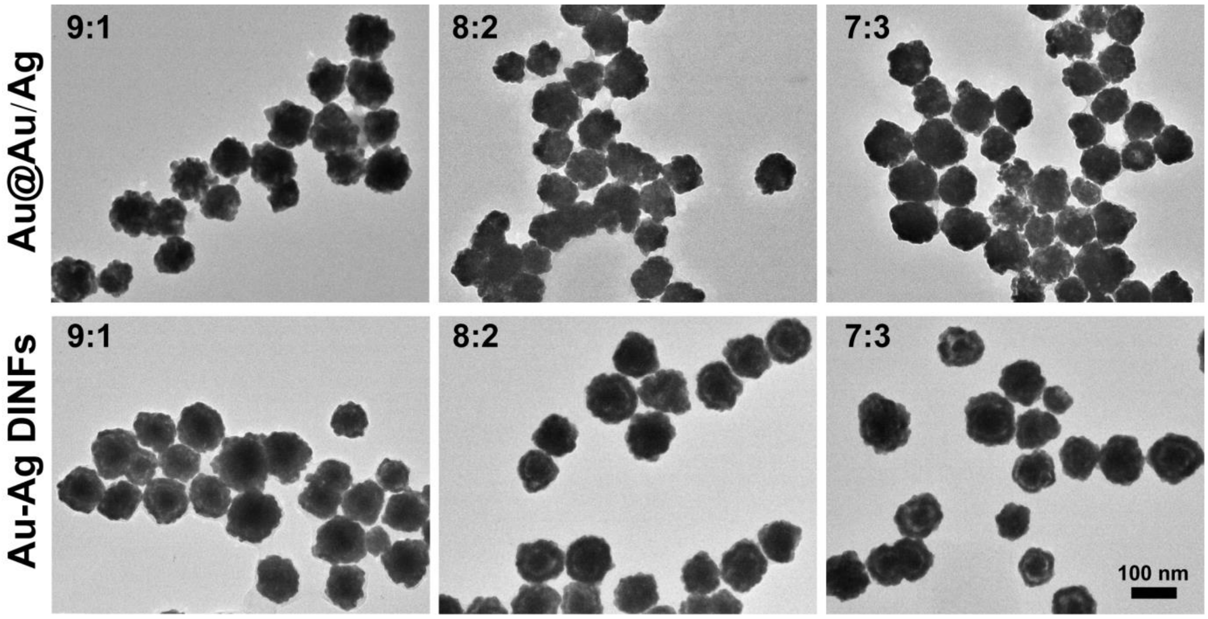

Figure S10. Representative TEM images of Au@Au/Ag and Au-Ag DINF fabricated with different ratio of Au/Ag (9:1, 8:2, 7:3).

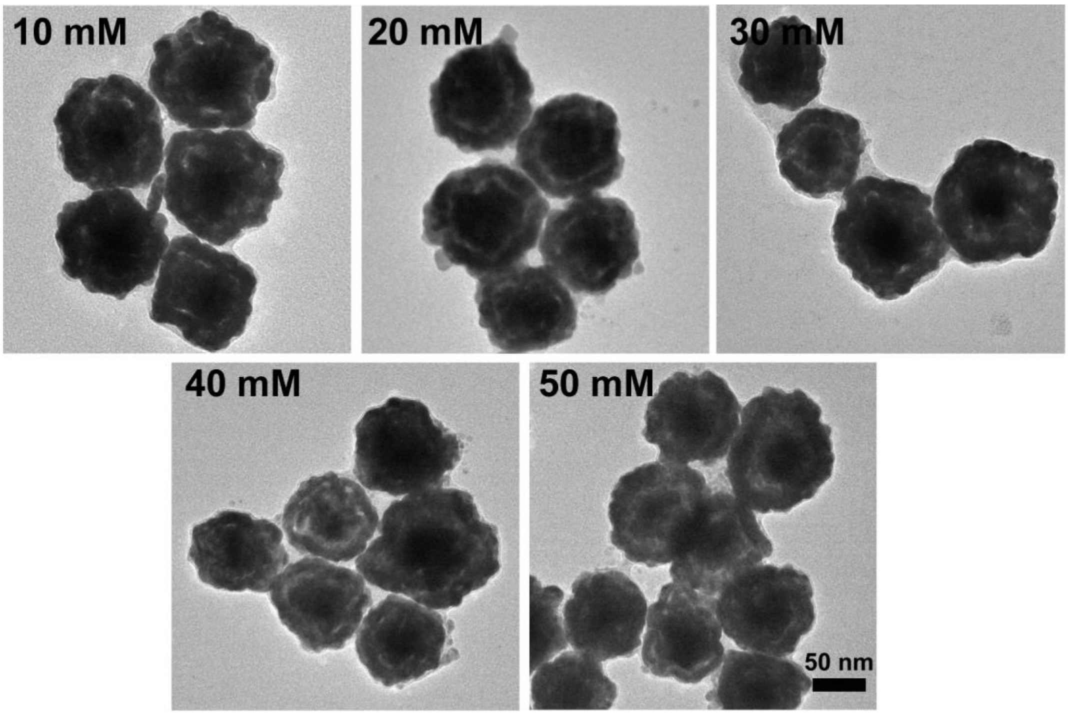

Figure S11. TEM images of Au-Ag DINF fabricated in different etching concentration (Au/Ag ratio = 8:2).

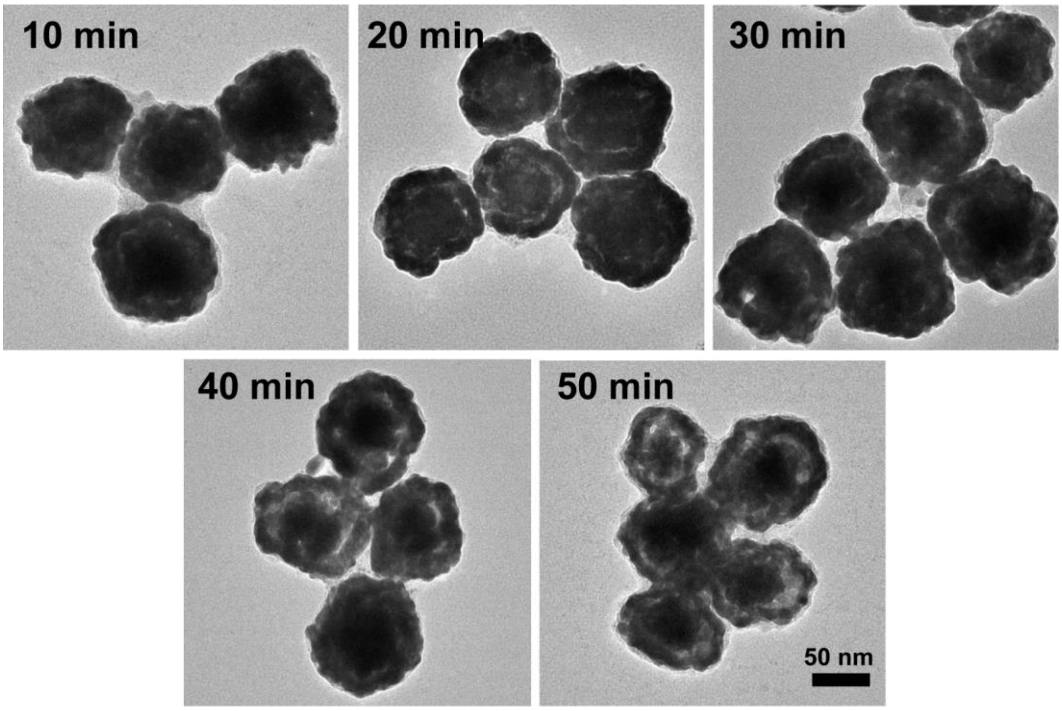

Figure S12. TEM images of Au-Ag DINF fabricated in different etching time (Au/Ag ratio = 8:2).

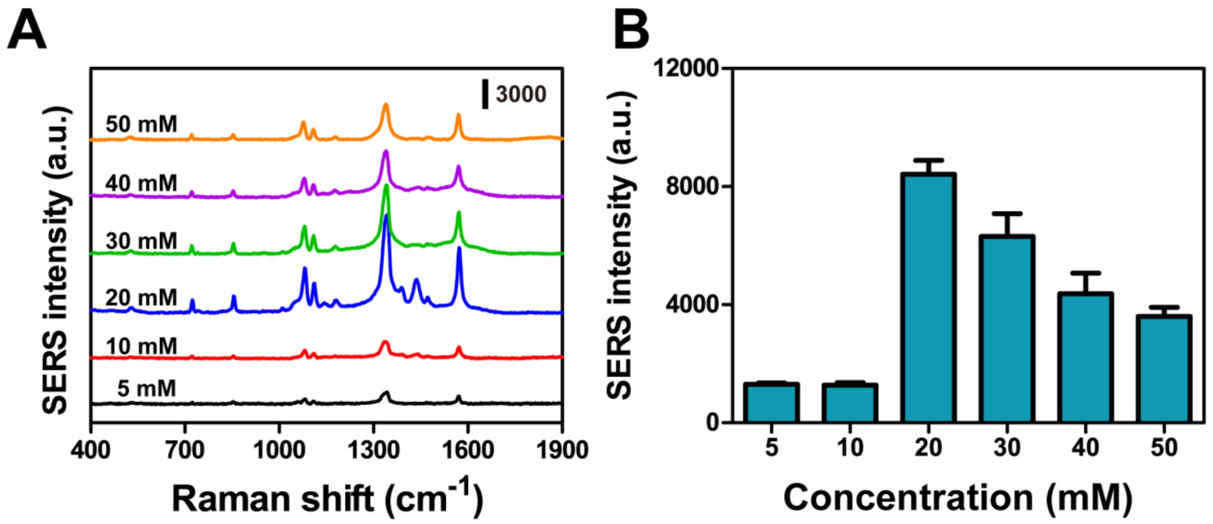

Figure S13. (A) SERS spectra of Au-Ag DINF fabricated in different etching concentration. (B) Corresponding SERS intensity at 1330 cm^{-1}. Data are presented as mean ± SD.

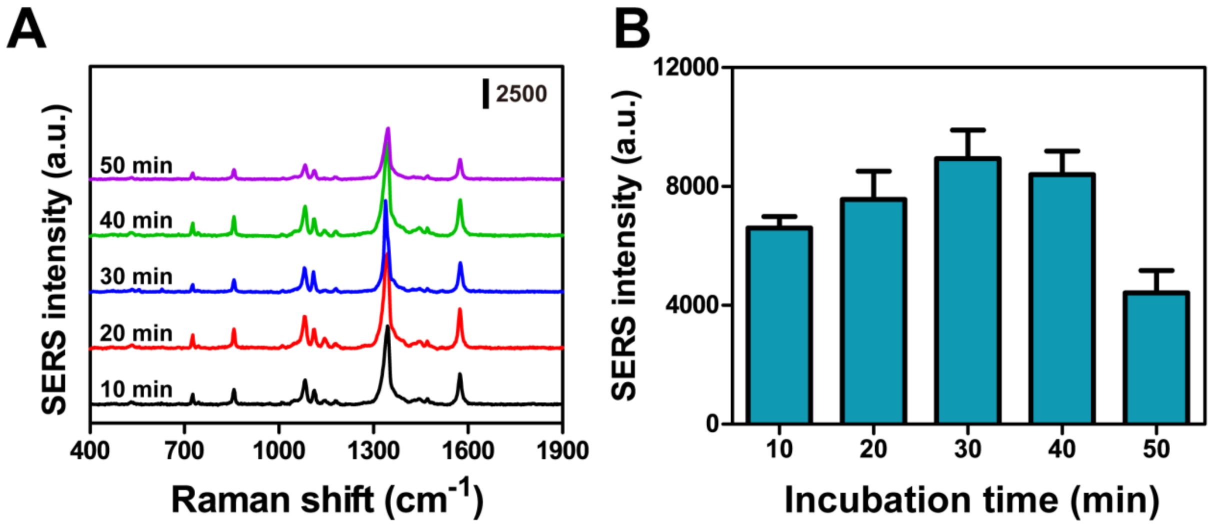

Figure S14. (A) SERS spectra of Au-Ag DINF fabricated in different etching time. (B) Corresponding SERS intensity at 1330 cm^{-1}. Data are presented as mean ± SD.

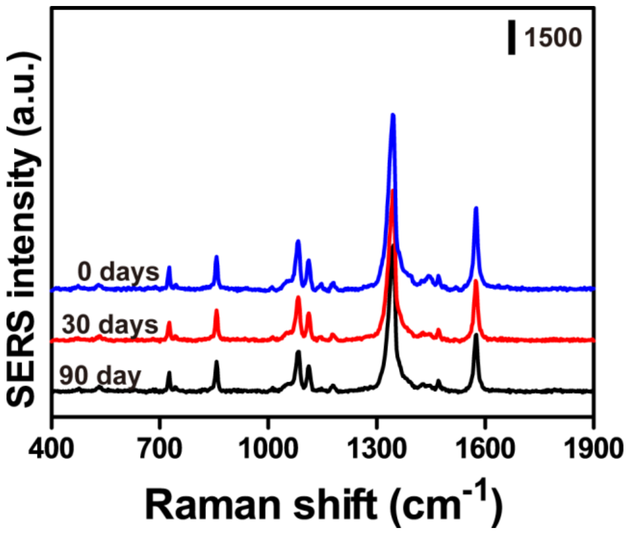

Figure S15. SERS spectra of Au-Ag DINF before and after stored at 4 °C for 0, 30 and 90 days.

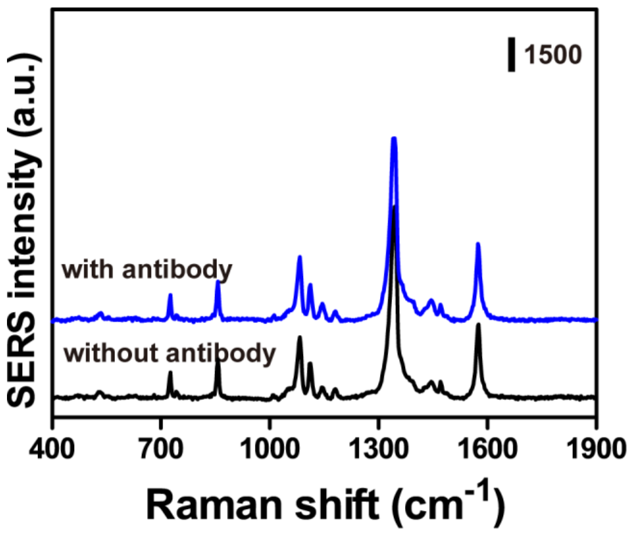

Figure S16. SERS spectra of Au-Ag DINF with or without Ab modification.

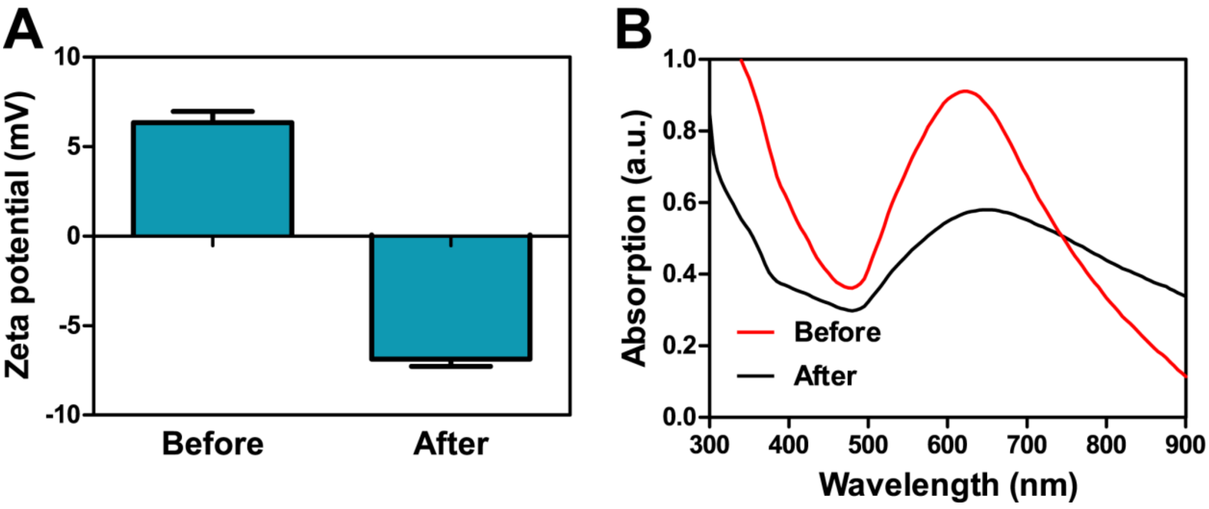

Figure S17. (A) Zeta potential and (B) UV-vis spectra of Au-Ag DINF before or after Ab modification. Data are presented as mean ± SD.

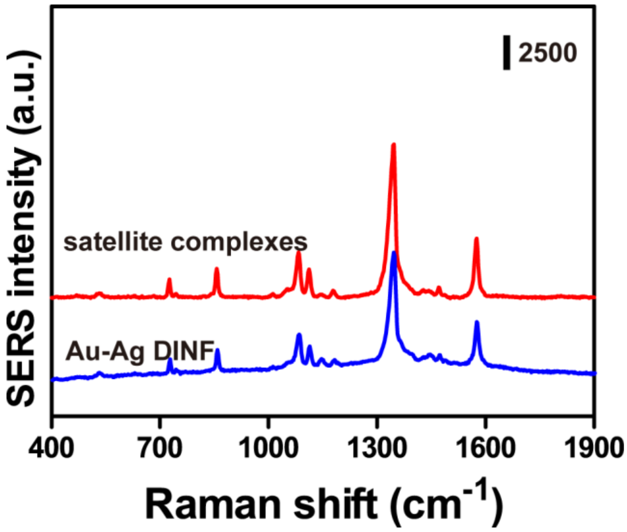

Figure S18. SERS spectra of Au-Ag DINF and Au-Ag DINF mediated satellite complexes.

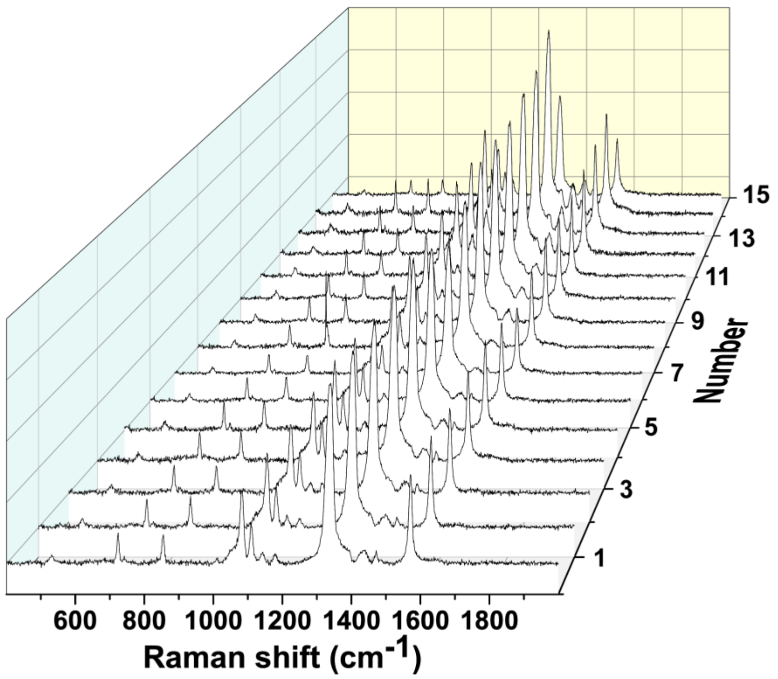

Figure S19. SERS performance of satellite complexes captured by AuMNPs from different area (*n=15*).

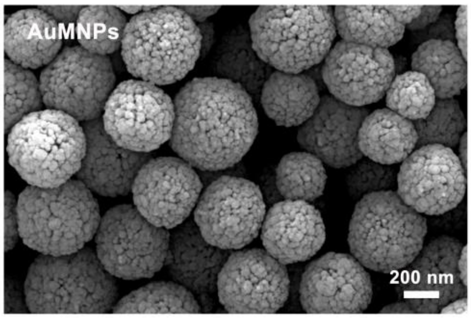

Figure S20. Representative SEM images of AuMNPs over a wide range.

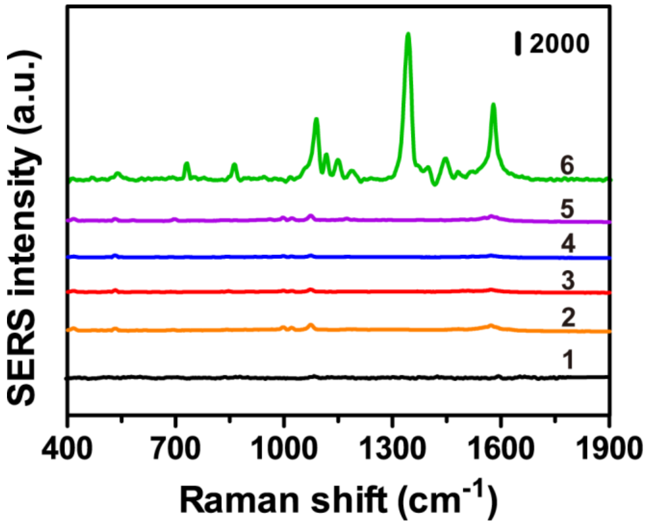

Figure S21. SERS spectra of different treated groups. From 1 to 6: Blank, PSA alone, Au-Ag DINF alone, Au-Ag DINF (+Ab) + PSA + AuMNPs (-Ab), Au-Ag DINF (-Ab) + PSA + AuMNPs (+Ab) and Au-Ag DINF (+Ab) + PSA + AuMNPs (+Ab) solutions.

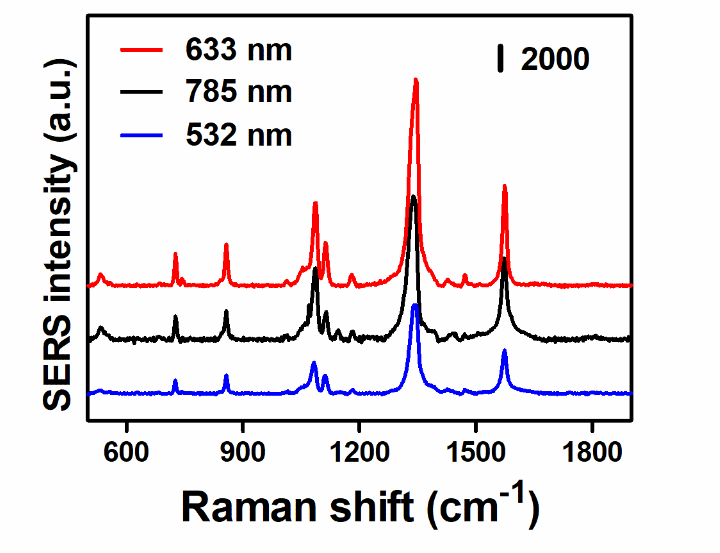

Figure S22. SERS spectra of Au-Ag DINF obtained from different excitation wavelengths (785 nm, 633 nm, 532 nm) with laser power at 0.5 mW.

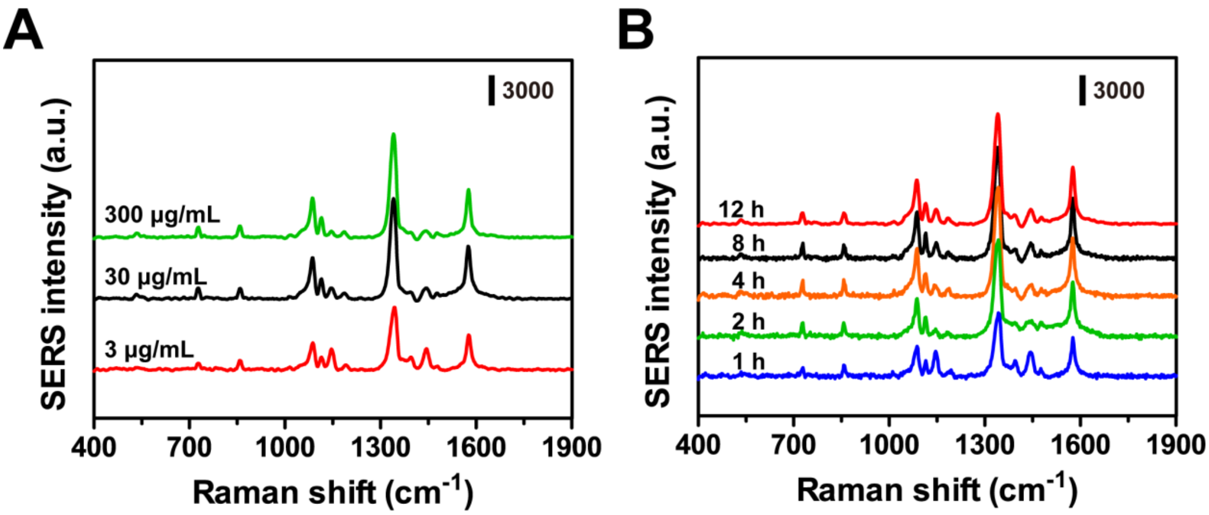

Figure S23. (A) SERS spectra of Au-Ag DINF modified with different concentration of Ab. (B) SERS spectra of Au-Ag DINF incubated with Ab in different time.

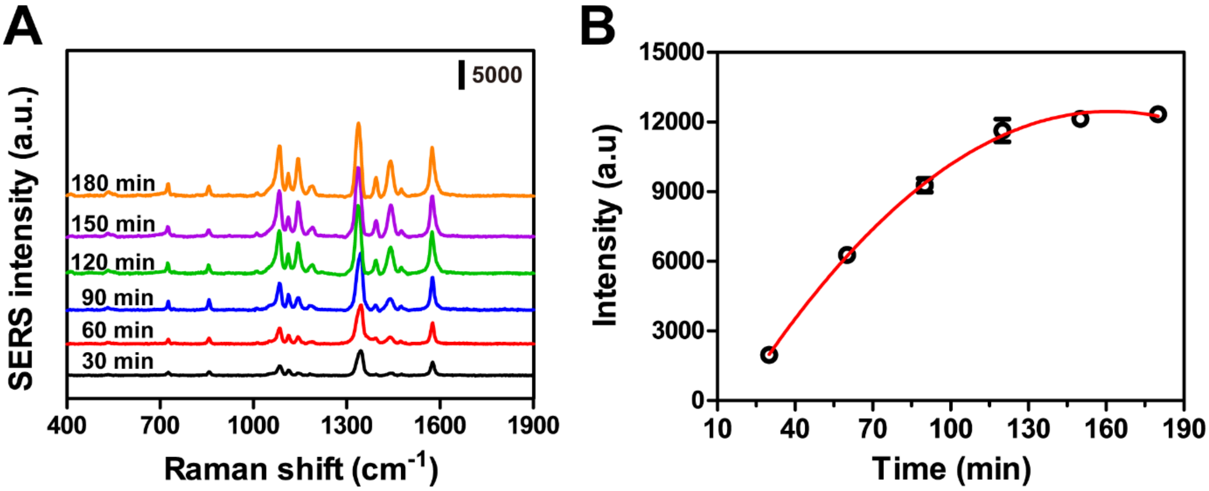

Figure S24. (A) Optimization the incubation time of satellite structure formation. (B) SERS intensity at 1330 cm^{-1} according to (A). Data are presented as mean ± SD.

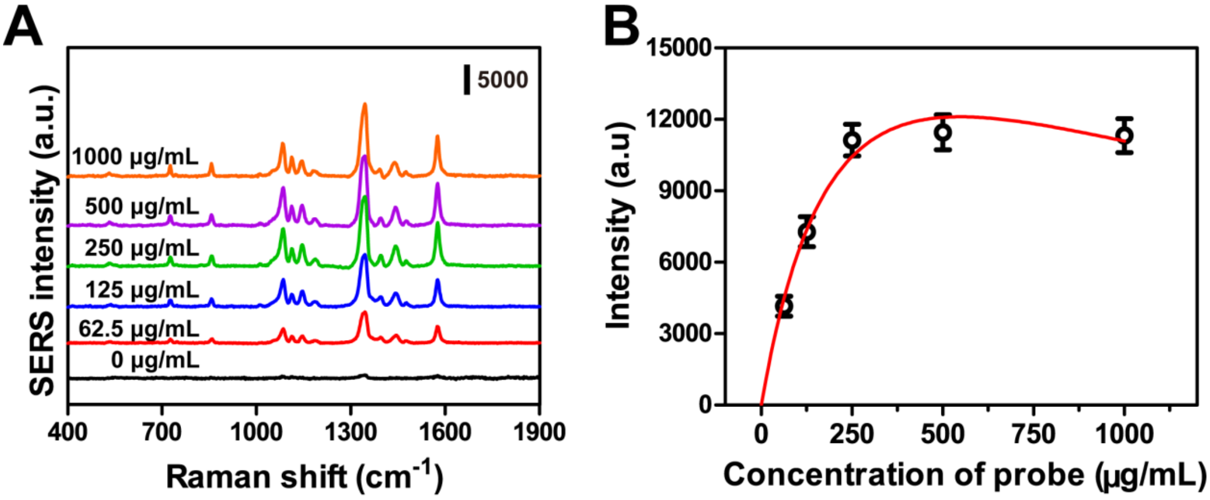

Figure S25. (A) Optimization the SERS probe concentration of satellite structure formation. (B) SERS intensity at 1330 cm^{-1} according to (A). Data are presented as mean ± SD.

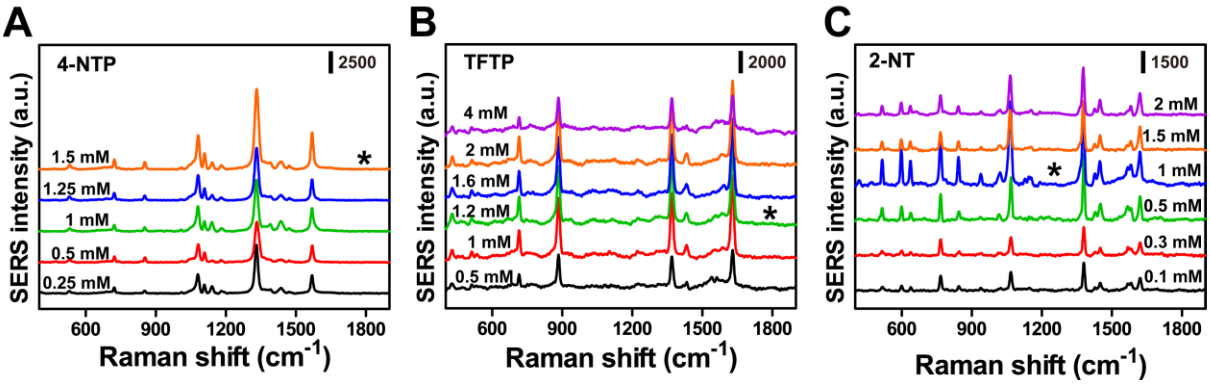

Figure S26. Optimization the concentration of different Raman dye. (A) for 4-NTP, (B) for TFTP, (C) for 2-NT.

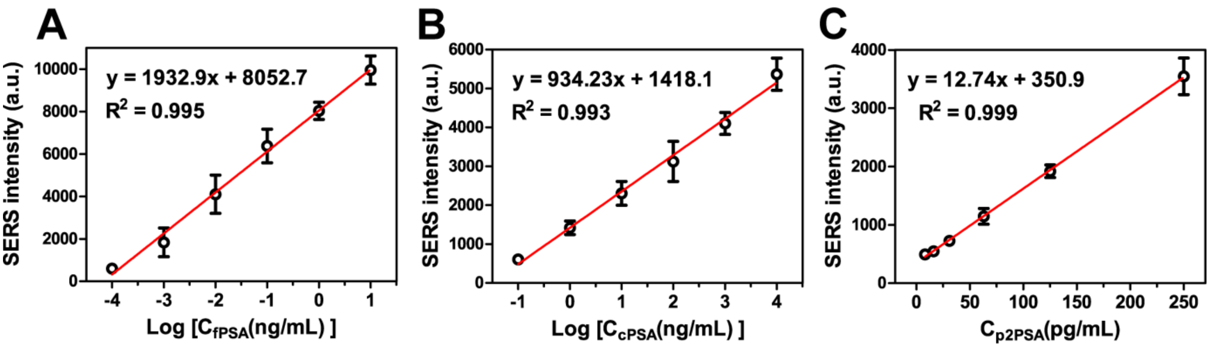

Figure S27. Standard curves of the SERS peak intensity at 1330, 881 and 767 cm^{-1} against the concentrations of (A) fPSA, (B) cPSA, and (C) p2PSA, respectively. Data are presented as mean ± SD.

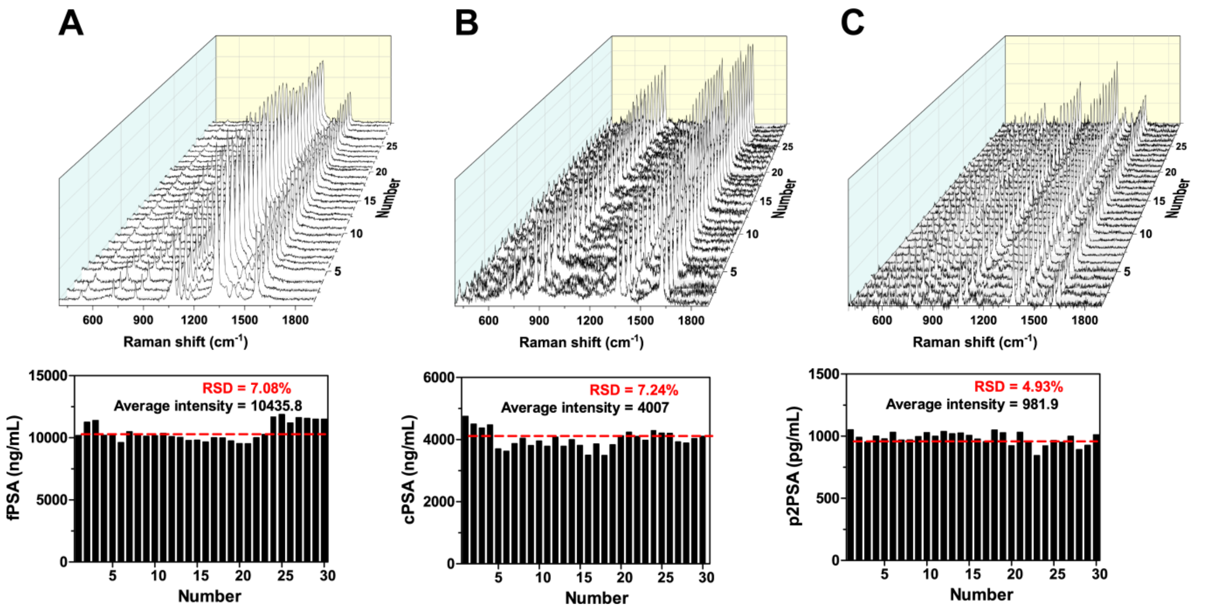

Figure S28. (A) 30 randomly selected SERS spectra acquired from Au-Ag DINF-mediated fPSA detection in different batch, and SERS intensity at 1330 cm^{-1} corresponding to (A) (RSD = 7.08 %). (B) 30 randomly selected SERS spectra acquired from Au-Ag DINF-mediated cPSA detection in different batch, and SERS intensity at 881 cm^{-1} corresponding to (A) (RSD = 7.24 %). (C) 30 randomly selected SERS spectra acquired from Au-Ag DINF-mediated p2PSA detection in different batch, and SERS intensity at 767 cm^{-1} corresponding to (A) (RSD = 4.93 %).

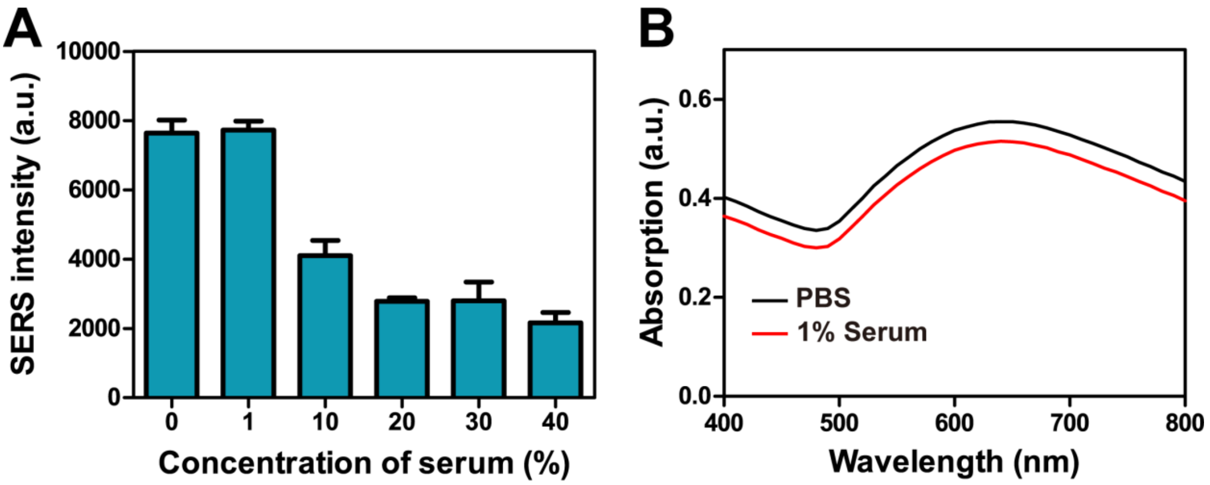

Figure S29. (A) Au-Ag DINF for the detection of fPSA in different diluted human blood samples. Data are presented as mean ± SD. (A) UV-vis absorption spectra of Au-Ag DINF in diluted blood samples (1%).

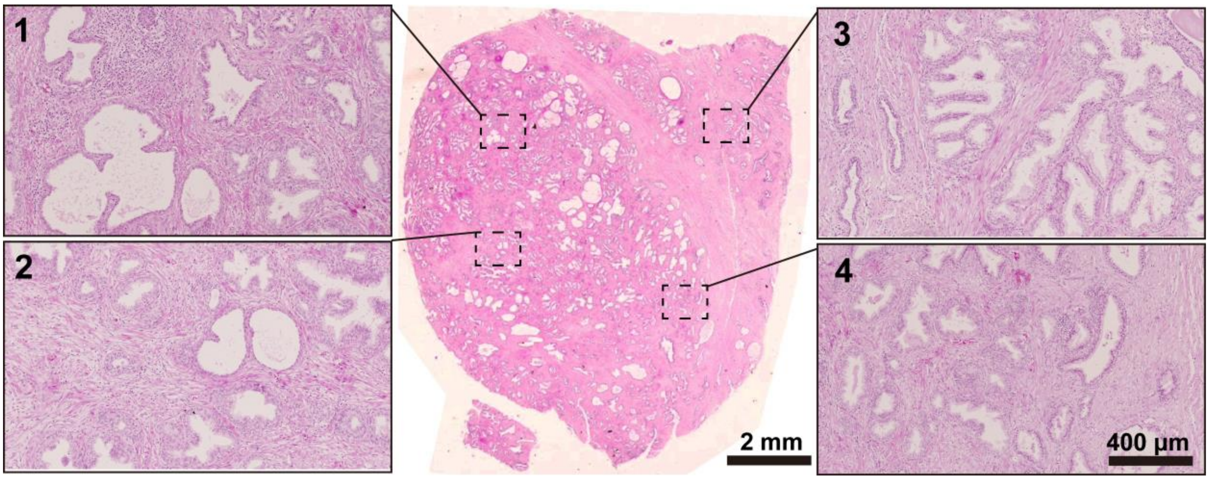

Figure S30. H&E staining of the suspicious tissues from P13 (patients 13), who was eventually identified as BPH or prostatitis rather than PCa. Points 1-4 indicated the magnified views in different areas.

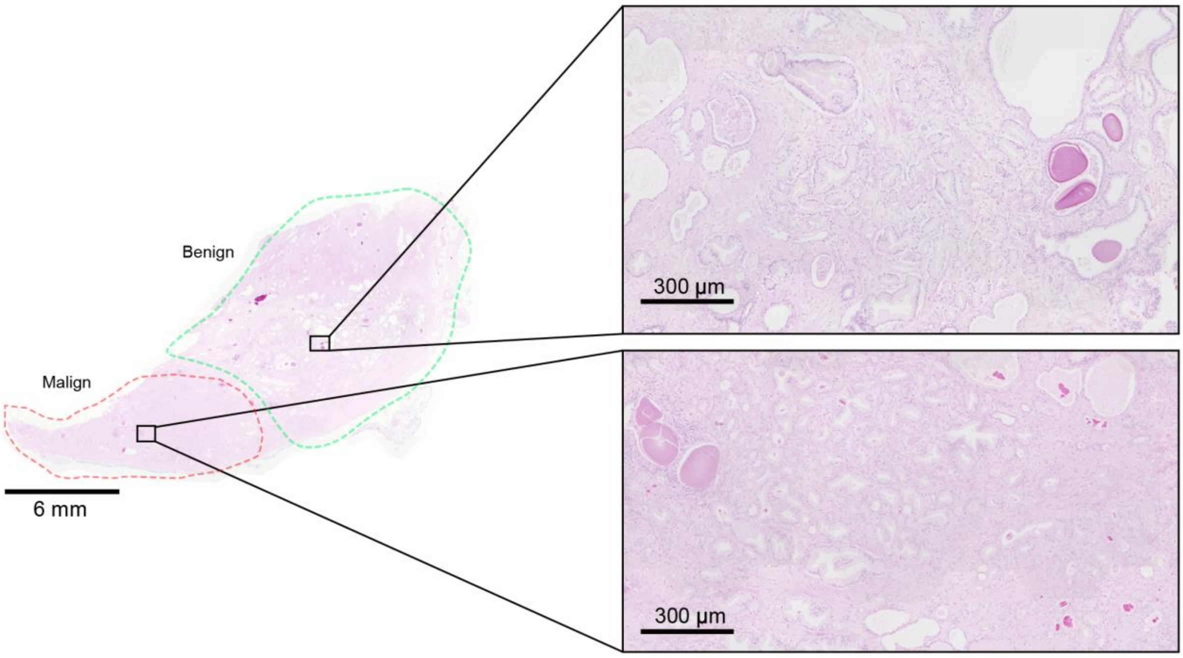

Figure S31. H&E staining of positive PCa sample (P6 patient). The red dotted lines represented areas of malignancy, and the green dotted lines represented areas of benign tissue.

Table S1. Comparison of the sensitivity of different analysis methods

Method	Linear ranges	LOD	Ref
SERS-based microfluidic analysis	0.05-100 ng/mL for fPSA<br>0.05-100 ng/mL for tPSA	0.1 ng/mL for fPSA<br>0.1 ng/mL for tPSA	[1]
Fluorescent analysis	0.64-80 ng/mL for fPSA<br>0.2-50 ng/mL for cPSA	22 pg/mL for fPSA<br>45 pg/mL for cPSA	[2]

SERS analysis	5.0 pg/mL-50 ng/mL for fPSA<br>45.0 pg/mL-450 ng/mL for cPSA	0.012 ng/mL for fPSA<br>0.15 ng/mL for cPSA	[3]
Fluorescent analysis	0.1-100 ng/mL for fPSA<br>0.1-100 ng/mL for cPSA	0.009 ng/mL for fPSA<br>0.087 ng/mL for cPSA	[4]
ECL analysis	1 pg/mL-10 ng/mL for tPSA	0.3 pg/mL for tPSA	[5]
Electrochemical analysis	0.75-100 ng/mL for tPSA	0.27 ng/mL for tPSA	[6]
Electrochemical analysis	1-10 ng/mL for fPSA<br>1-10 ng/mL for tPSA	1 ng/mL for fPSA<br>1 ng/mL for tPSA	[7]
Chemiluminescent analysis	0.01-36.7 ng/mL for fPSA<br>0.02-125 ng/mL for tPSA	0.004 ng/mL for fPSA<br>0.007 ng/mL for tPSA	[8]
This method	10^{-4}-10 ng/mL for fPSA<br>10^{-1}-10^{4} ng/mL for cPSA<br>0.008-0.25 ng/mL for p2PSA	0.11 pg/mL for fPSA<br>0.12 ng/mL for cPSA<br>0.004 ng/mL for p2PSA	

Table S2. Recovery results of PSA content in human serum. (N = 3/group)

Sample no	Spiked concentration	Detected concentration	Recovery (%)	RSD (%)
fPSA (ng/mL)	0.5	0.51	102	6.0
	5	5.13	102.6	7.3
	10	10.44	104.4	4.2
cPSA (ng/mL)	0.5	0.49	98.0	5.3
	5	5.38	107.6	2.2
	10	10.27	102.7	7.8
p2PSA (pg/mL)	0.5	0.56	112	7.4
	5	5.32	106.4	3.3
	10	11.11	111.1	9.1

Table S3. Clinical background of PCa patients and the results of PSA assay.

Sample no	Sex	age	Clinical assay		SERS-based assay	
			fPSA (ng/mL)	cPSA (ng/mL)	fPSA (ng/mL)	cPSA (ng/mL)
H1	M	68	0.06	0.10	0.053	0.094
H2	M	58	0.01	0.01	0.010	0.020

H3	M	73	0.43	2.11	0.404	2.211
H4	M	74	0.15	1.68	0.152	1.746
H5	M	*N.A.*	0.12	0.34	0.109	0.309
P6	M	54	0.43	4.89	0.422	5.371
P7	M	75	0.86	6.86	0.792	5.595
P8	M	79	0.53	4.29	0.482	3.949
P9	M	71	0.86	9.34	0.934	9.314
P10	M	*N.A.*	0.49	10.21	0.377	9.752
P11	M	67	1.32	7.46	1.128	6.437
P12	M	78	1.34	4.97	1.396	4.189
P13	M	55	1.47	4.87	1.134	5.479
P14	M	70	2.29	7.81	2.507	7.467
P15	M	53	1.52	5.45	1.424	5.619
P16	M	*N.A.*	8.39	32.01	7.944	31.802
P17	M	*N.A.*	2.80	40.00	2.882	40.538
P18	M	*N.A.*	1.17	27.63	1.097	29.631
P19	M	*N.A.*	6.62	17.48	6.038	17.921
P20	M	75	5.72	25.98	5.419	25.578
P21	M	75	3.21	48.59	3.029	49.769
P22	M	60	1.22	16.18	1.062	17.804
P23	M	77	2.23	18.17	2.421	17.813

Notice: *N.A.*= not applicable.

References

[1] R. Gao, Z. Cheng, X. Wang, L. Yu, Z. Guo, G. Zhao, J. Choo, *Biosens. Bioelectron.* **2018**, *119*, 126.

[2] X. Min, S. Huang, C. Yuan, *Anal. Chim. Acta* **2022**, *1204*, 339704.

[3] Z. Cheng, N. Choi, R. Wang, S. Lee, K.C. Moon, S.Y. Yoon, L. Chen, J. Choo, *ACS Nano* **2017**, *11*, 4926.

[4] Z. Rong, Z. Bai, J. Li, H. Tang, T. Shen, Q. Wang, C. Wang, R. Xiao, S. Wang, *Biosens. Bioelectron.* **2019**, *145*, 111719.

[5] M.S. Khan, W. Zhu, A. Ali, S.M. Ahmad, X. Li, L. Yang, Y. Wang, H. Wang, Q. Wei, *Anal. Biochem.* **2019**, *566*, 50.

[6] C. Thunkhamrak, P. Chuntib, K. Ounnunkad, P. Banet, P.H. Aubert, G. Saianand, A.I. Gopalan, J. Jakmunee, *Talanta*. **2020**, *208*, 120389.

[7] V. Escamilla-Gómez, D. Hernández-Santos, M.B. González-García, J.M. Pingarrón-Carrazón, A. Costa-García, *Biosens. Bioelectron.* **2009**, *24*, 2678.
[8] A. Liu, F. Zhao, Y. Zhao, L. Shangguan, S. Liu, *Biosens. Bioelectron.* **2016**, *81*, 97.
